# From Pinocytosis to Methuosis—Fluid Consumption as a Risk Factor for Cell Death

**DOI:** 10.3389/fcell.2021.651982

**Published:** 2021-06-23

**Authors:** Markus Ritter, Nikolaus Bresgen, Hubert H. Kerschbaum

**Affiliations:** ^1^Center for Physiology, Pathophysiology and Biophysics, Institute for Physiology and Pathophysiology, Paracelsus Medical University, Salzburg, Austria; ^2^Institute for Physiology and Pathophysiology, Paracelsus Medical University, Nuremberg, Germany; ^3^Gastein Research Institute, Paracelsus Medical University, Salzburg, Austria; ^4^Ludwig Boltzmann Institute for Arthritis und Rehabilitation, Salzburg, Austria; ^5^Kathmandu University School of Medical Sciences, Dhulikhel, Nepal; ^6^Department of Biosciences, University of Salzburg, Salzburg, Austria

**Keywords:** pinocytosis, macropinocytosis, endocytosis, intracellular vesicle, ion transport, cell volume regulation, cell death, methuosis

## Abstract

The volumes of a cell [cell volume (CV)] and its organelles are adjusted by osmoregulatory processes. During pinocytosis, extracellular fluid volume equivalent to its CV is incorporated within an hour and membrane area equivalent to the cell’s surface within 30 min. Since neither fluid uptake nor membrane consumption leads to swelling or shrinkage, cells must be equipped with potent volume regulatory mechanisms. Normally, cells respond to outwardly or inwardly directed osmotic gradients by a volume decrease and increase, respectively, i.e., they shrink or swell but then try to recover their CV. However, when a cell death (CD) pathway is triggered, CV persistently decreases in isotonic conditions in apoptosis and it increases in necrosis. One type of CD associated with cell swelling is due to a dysfunctional pinocytosis. Methuosis, a non-apoptotic CD phenotype, occurs when cells accumulate too much fluid by macropinocytosis. In contrast to functional pinocytosis, in methuosis, macropinosomes neither recycle nor fuse with lysosomes but with each other to form giant vacuoles, which finally cause rupture of the plasma membrane (PM). Understanding methuosis longs for the understanding of the ionic mechanisms of cell volume regulation (CVR) and vesicular volume regulation (VVR). In nascent macropinosomes, ion channels and transporters are derived from the PM. Along trafficking from the PM to the perinuclear area, the equipment of channels and transporters of the vesicle membrane changes by retrieval, addition, and recycling from and back to the PM, causing profound changes in vesicular ion concentrations, acidification, and—most importantly—shrinkage of the macropinosome, which is indispensable for its proper targeting and cargo processing. In this review, we discuss ion and water transport mechanisms with respect to CVR and VVR and with special emphasis on pinocytosis and methuosis. We describe various aspects of the complex mutual interplay between extracellular and intracellular ions and ion gradients, the PM and vesicular membrane, phosphoinositides, monomeric G proteins and their targets, as well as the submembranous cytoskeleton. Our aim is to highlight important cellular mechanisms, components, and processes that may lead to methuotic CD upon their derangement.

## Introduction

Individual cells have the same need entire organisms have: They have to drink. At the cellular level, water drinking is known as pinocytosis and the fluid-containing organelle is the pinosome. Fluid uptake, intracellular distribution, and processing require precise spatial and temporal coordination of membrane and cytoskeletal proteins. Macropinocytosis is an actin-driven process, where a cup-like structure emerges from the cell surface, which engulfs extracellular fluid and forms a vesicle (Swanson and Watts, 1995; Swanson, 2008; Kerr and Teasdale, 2009; Lim and Gleeson, 2011; Hinze and Boucrot, 2018; Doodnauth et al., 2019; King and Kay, 2019; Swanson and King, 2019). The vesicle membrane contains ion channels and transporters that are used for the flux of water, osmolytes, and nutrients, and it is decorated by distinct phospholipids and proteins that are required for intracellular sorting and transport of the organelle (Bohdanowicz and Grinstein, 2013; Levin et al., 2015; Freeman and Grinstein, 2018; Williamson and Donaldson, 2019; Stow et al., 2020) (Table 1). Macropinocytosis serves seemingly unrelated cellular functions, such as nutrition acquisition to satisfy cellular energy demands (Recouvreux and Commisso, 2017; Palm, 2019; Lin et al., 2020), immune surveillance leading to antigen presentation to lymphocytes (Lanzavecchia, 1996; Von Delwig et al., 2006; Liu and Roche, 2015; Canton, 2018), intracellular replication of pathogenic bacteria (Bloomfield and Kay, 2016; Di Russo Case et al., 2016), and CD by drinking too much fluid, i.e., methuosis (Maltese and Overmeyer, 2014, 2015). Careful examination of these functions showed similarities in the initial steps of fluid uptake and differences in the final processing steps, such as fusion or not fusion with lysosomes.

**TABLE 1:** Some characteristics and ion concentrations of macropinocytosis and the endolysosomal pathway.

		Appearance	PIPs	Decoration	Ψ_m_ (mV)	pH	Na^+^ (mM)	K^+^ (mM)	Cl^–^ (mM)	HCO_3_^–^ (mM)	Ca^2+^ (mM)
ECF NP	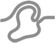	Irregular	PI(4,5)P2	Rab5, Rab20	∼−30 to −70	7.4	120–150	4–5	110–120	24–27	2
ICF						6.9	12	140	20–80	8–15	0.1–2
EE	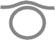	Spherical	PI3P, PI(3,4)P2	Rab4, Rab5, Rab20	10–20	6.0–6.2			20–30		0.003–2
LE	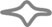	Tubulated	PI3P, PI(3,5)P2	Rab7, Rab9, Rab20		5.0–5.5	20		40–70		2.5
MVB	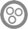	Intraluminal vesicles	PI3P + PI(3,5)P2	Rab7, Rab9, Rab20		5.5			40		0.009
LE/LY Hybrid	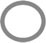		PI3P	Rab,7, LAMP1							
LY	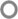	Spherical	PI(3)P + PI(3,5)P2	LAMP1	∼−40 to +20	4.3–5.5	20–145	2–60	>80		0.4–0.7

*ECF, extracellular fluid; ICF, intracellular fluid; NP nascent pinosome; EE, early endosome; LE, late endosome; MVB, multivesicular body; LY, lysosome. For details, see text. References: (Sonawane and Verkman, 2003; Dong et al., 2010; Morgan et al., 2011; Scott and Gruenberg, 2011; Welliver and Swanson, 2012; Bohdanowicz and Grinstein, 2013; Ishida et al., 2013; Stauber and Jentsch, 2013; Chang et al., 2014; Egami et al., 2014; Levin et al., 2015; Saha et al., 2015; Xu and Ren, 2015; Xiong and Zhu, 2016; Chakraborty et al., 2017; Kim et al., 2018; Sterea et al., 2018; King and Kay, 2019; Li P. et al., 2019; Adjemian et al., 2020; Jin et al., 2020; Trivedi et al., 2020; Chadwick et al., 2021b; Riess et al., 2021; Zhang et al., 2021).*

**FIGURE 1:**
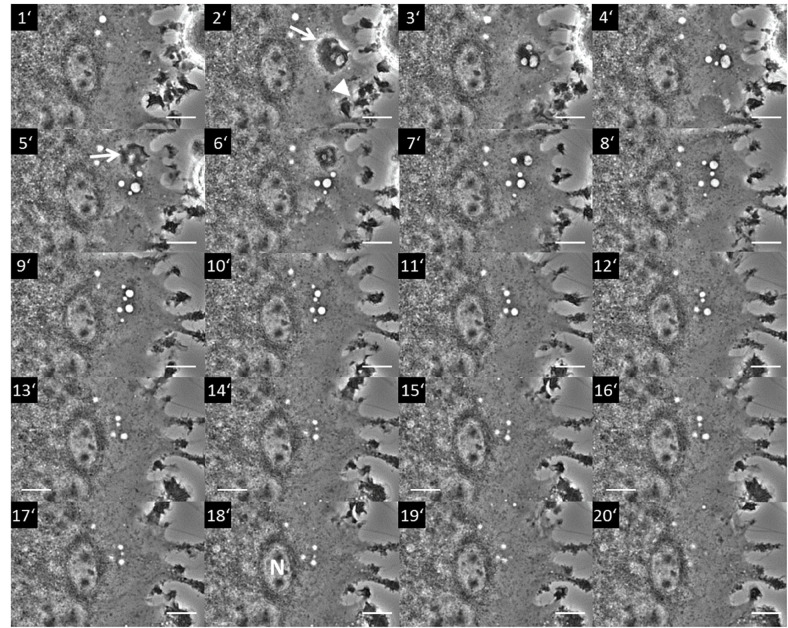
Macropinocytosis occurring in a section of a multinucleated giant cell from a rat non-parenchymal hepatic cell line putatively representing immortalized monocytes (Kupffer cells). The image series shows a section of a giant cell where pinocytosis occurs. The process starts with the formation of a membrane ruffle at minute 2 (2′; arrow) from which an array of vesicles (pinosomes) originates (3′–4′). Further ruffling can be seen at the cell periphery (2′; arrowhead). A second ruffle is forming at minute 5 (5′; arrow) from which additional pinosomes derive (5′–7′). The whole pinosome array subsequently moves toward the center area of the giant cell (8′–18′) locating to the vicinity of a nucleus (N in 18′). Terminally, the vesicles start to disappear in the perinuclear (pericentral) cytosol (20′). Scale bar = 10 μm. The whole live cell imaging sequence can be seen in Supplementary Video 1.

The prototypic experiments examining pinocytosis were done on macrophages and malignant cells by Lewis in the 1930s (Lewis, 1936, 1937). Then, Lewis was among the few researchers using time-lapse microscopy to study the dynamics of living cells. Lewis introduced the term “*pinocytosis*” to describe the fact that ruffle formation is associated with vesicle formation and uptake of extracellular fluid (Lewis, 1936, 1937). In line with Lewis’s statement “*Pinocytosis is easily seen in motion pictures*” (Lewis, 1937), we use video microscopy to visualize the dynamics of pinocytosis (Figure 1 and Supplementary Videos 1, 2). In the words of Lewis, Supplementary Video 1 shows that vesicles “*taken in vary greatly in size*,” “*several fuse*… *to form larger ones*,” “*move centrally*,” and “*finally reach*… *the neighborhood of*… *the nucleus*” (Lewis, 1937). Lewis also suggested that proteins in the vesicles are “*split by the digestive enzymes into simpler products which can be utilized or can diffuse out of the cell*.” He also related the “*disappearance*” of the vesicles “*with the completion of the digestion of their contents*” as the vesicles “*slowly shrink in size and disappear, leaving a small granule*…” (Lewis, 1937). Lewis’s experiments also showed that the cells do not increase in volume, although “*they may take in several times their volume of fluid*” and he “*assumed that the fluid diffuses out of the cells when the globules disappear*” (Lewis, 1937). These experiments demonstrate that volume regulatory processes at the cellular and organelle levels are of paramount importance to maintain CV while cells incorporate large volumes of extracellular fluid and digest macromolecules present in the fluid. The fact that the fluid taken up by macropinocytosis outweighs its elimination by recycling vesicles (Swanson, 1989; Choy et al., 2018) highlights that VVR and CVR are inevitably linked to each other. This becomes evident from the massive cell swelling seen upon inhibition of water channels in pinocytosing cells (De Baey and Lanzavecchia, 2000). Furthermore, considering that the total volume of the endolysosomal compartment can make up a substantial part of the total CV (Choy et al., 2018) makes evident that the exchange of osmotically active solutes between the endolysosomal compartment and the cytosol will strictly affect the volumes of either part.

Given the importance of CVR, a central question is: What determines the size of a vesicle? That is, which transporters and ion channels in the PM and vesicle membrane are activated, incorporated, and terminated at which spatial and temporal check points? How does the macropinosomal solute composition and the vesicular membrane properties change after having gulped a lot of extracellular fluid during maturation along the endolysosomal pathway, and what are the determinants of this change? And finally: How are these cellular and subcellular volume regulatory mechanisms altered in methuosis?

To understand subcellular volume regulation, findings related to CVR provide clues to identify factors maintaining the set points of the vesicle.

As ion channels and transporters in the PM are central in CVR, they also contribute to VVR (Freeman and Grinstein, 2018; Freeman et al., 2020; Chadwick et al., 2021b). Thus, Lewis’s statement in the 1930s that “*The factors involved in the diffusion of the fluid out of the cell are as mysterious as most of the other processes which take place*” (Lewis, 1937) is now transforming to hypotheses trying to explain CVR and VVR in macropinocytosis at the molecular level and by facts generated by electrophysiological, molecular–biological, and imaging studies. Notably, Freeman and Grinstein (2018); Freeman et al. (2020), and Chadwick et al. (2021b) demonstrated that contributions from ion transporters are essential for normal shrinkage in macropinosome maturation.

This review focuses on the complex mutual interplay between extracellular and intracellular ions and ion gradients, the PM and vesicular membrane, phosphoinositides, monomeric G proteins and their targets, as well as the submembranous cytoskeleton in pinocytosis, with special emphasis on its connection to CVR and VVR. It aims at highlighting important cellular mechanisms and components that govern these processes and that may lead to methuotic cell death upon their derangement.

**FIGURE 2:**
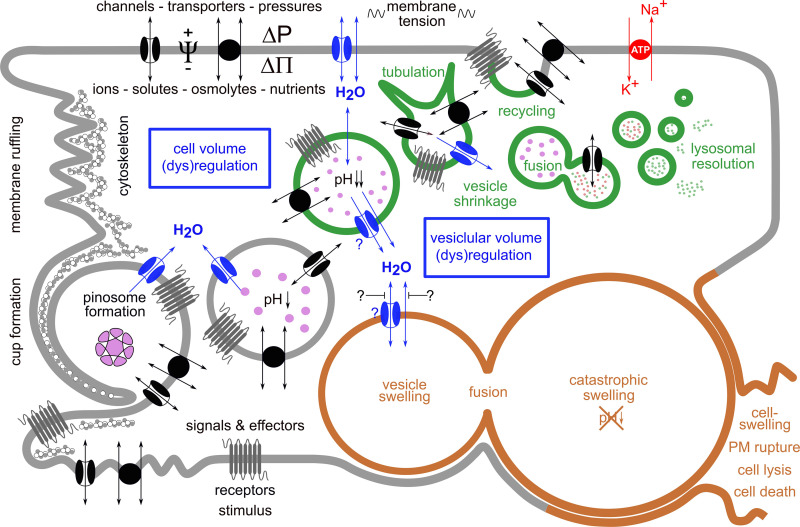
Simplified scheme of key processes in vesicle/vacuole formation and processing during normal macropinocytosis and in methuosis. Macropinocytosis is an actin-driven process that is triggered by various stimuli. It starts with ruffling of the plasma membrane (PM) and formation of a pinocytotic cup, which engulfs extracellular fluid and forms a pinosome by membrane fusion at the tip of a lamellipodium-like structure. Its membrane contains ion channels and transporters, receptors, among other various PM constituents, e.g., phospholipids. The nascent pinosome unselectively engulfs extracellular fluid, along with its ions, nutrients, and metabolites and eventually also toxins or drugs. It may also enclose particulate matter-like exosomes, micro- or nanoparticles, or pathogens such as bacteria and viruses (pink enclosed structure). Under normal conditions (green vesicles), pinosomes move centripetally, become more and more acidic, shrink along their route, and become tubulated, a process necessary for proper sorting and recycling of the vesicles and their cargo. This requires also its decoration with distinct phospholipids and proteins (not shown). Reusable membrane proteins may become inserted again into the PM when vesicles fuse with it. This also recycles incorporated membrane back to the PM and relieves its tension. Vesicles designated for delivery of their contents to lysosomes—for further processing, digestion, or destruction—fuse with them and finally resolve. The resulting products may be further used, e.g., for the cell’s nutrition. To ensure these processes, cell volume regulation and vesicular volume regulation must work hand in hand. This becomes evident from the fact that during pinocytosis, an extracellular fluid volume and membrane area equivalent to the cell’s volume and to the cell’s surface are incorporated within 1 h and 30 min, respectively. The volume regulatory mechanisms involve movement of ions and osmolytes across the PM by means of specific ion channels and transporters. The driving forces for these fluxes are determined by the electrochemical gradients. Water flux is driven mainly by the osmotic (ΔΠ) but eventually also by the hydrostatic (ΔP) pressure differences. The water permeability of the PM is greatly enhanced by water channels (aquaporins, blue). All of these movements are primarily driven by the activity of the Na^+^/K^+^-ATPase (red transporter in the PM), an ion pump that moves Na^+^ ions out of and K^+^ ions into the cell, while hydrolyzing ATP as energy source. This process sets up the required ionic and osmotic gradients and determines the electrical potential difference Ψ across the PM. The mechanisms for vesicular volume regulation follow the same rules and are in principle identical to those in cell volume regulation, while utilizing their individual set of transporters and channels. In methuosis, a lethal process of aberrant pinocytosis where cells “drink themselves to death,” these processes are severely disturbed (brown vesicles). Fluid uptake by macropinocytosis is enhanced, and instead of shrinking, the vesicles swell, do not acidify, remain non-functional, and do not fuse with lysosomes, but instead they with each other. This leads to the formation of giant vacuoles, catastrophic cell swelling, and consequently to rupture of the PM, cell lysis, and death.

Figure 2 schematically shows key steps of vesicle/vacuole formation and processing during normal macropinocytosis and in methuosis.

## Macropinocytosis

Macropinocytosis is a form of clathrin-independent endocytosis. Ruffling of cholesterol-rich membrane microdomains leads to the unselective incorporation of large volumes of extracellular fluid in macropinosomes with a diameter of 0.2 up to 5.0 μm (Levin et al., 2015; Donaldson, 2019). Macropinocytosis may occur constitutively or be induced by growth factors, chemokines, microbial products, viruses (Freeman et al., 2014; Marques et al., 2017; Canton, 2018; Doodnauth et al., 2019; Tejeda-Munoz et al., 2019), crosslinking of cell surface molecules (Imamura et al., 2011), knockdown of genes (Choi et al., 2017; Fomin et al., 2018; Fomin, 2019; Su et al., 2021), and constitutive expression (Kasahara et al., 2007) or mutations of signal transduction molecules (Yoo et al., 2020). Some cells, like innate immune cells and Ras-transformed cancer cells, are able to perform both forms of macropinocytosis (Amyere et al., 2000; Stow et al., 2020). Importantly, this process ensures that cells incorporate everything animals ingest and digest, including nutrients as well as toxic substances and metabolites released by neighboring cells, exosomes, microparticles, and pathogens, such as bacteria and viruses (Suda et al., 2007; Mercer and Helenius, 2009, 2012; Mercer et al., 2010; Commisso et al., 2013; Bloomfield and Kay, 2016; Jiang et al., 2017; Commisso, 2019). Furthermore, macropinocytosis is involved in cell migration (Wen et al., 2016; Swanson and King, 2019).

Remarkably, within 1 h, a volume equivalent of the entire cytoplasm is incorporated by macropinocytosis, and within 30 min, macropinosome formation requires the entire cellular PM surface (Steinman et al., 1976; Cullen and Steinberg, 2018; Freeman and Grinstein, 2018). Against the compelling background of conserved CV and cell surface, sorting mechanisms distinguishing between recycling and digestion routes are of eminent importance. Membrane recycling is not only of importance for maintaining the cell surface but also for supplying the PM with receptors and transporters, such as neonatal Fc-receptor (Toh et al., 2019), bone morphogenetic protein receptor (Kim et al., 2019), PDGF β-receptor (Schmees et al., 2012), EGF receptor (Chiasson-Mackenzie et al., 2018; Freeman et al., 2020), and other plasmalemmal components such as integrins (Buckley et al., 2016; Freeman et al., 2020) as well as small GTPases, which fuel macropinocytosis (Cullen and Steinberg, 2018). Furthermore, as the intercellular volume is usually small, changes in extracellular ion composition may greatly affect ion gradients, which drive nutrient transporters, e.g., Na^+^-dependent glucose or amino acid uptake (Ganapathy et al., 2008; Wright et al., 2011; Broer, 2014). This exceptional well-balanced system is keeping CV and cell surface during macropinocytic flow reasonably constant, but puts the cell at risk to damage either when CVR fails or when it accumulates metabolic waste products as seen in lysosomal storage diseases (Platt et al., 2012, 2018; Rappaport et al., 2016). Excessive fluid uptake in *in vitro* conditions leading to a distinct form of CD has been recently recognized and named methuosis (Maltese and Overmeyer, 2014, 2015).

### Ruffle and Cup Formation

Ruffle and cup formation is an actin-driven process, which is closely related to the formation of phagocytic cups and pseudopods (Lim and Gleeson, 2011; Freeman and Grinstein, 2014; Buckley and King, 2017; Williamson and Donaldson, 2019). Actin cytoskeleton rearrangement depends on phospholipids, lipid kinases and phosphatases, small GTPases, actin-modulating proteins, ion channels, and proton (H^+^) pumps (Welliver and Swanson, 2012). Among the numerous small GTPases, the Ras and Rho family member, Rac1, is critical in the formation of ruffles and macropinocytic cups (Egami et al., 2014; Donaldson, 2019). For small GTPases, the switch from an inactive guanosine diphosphate (GDP)-bound form to an active guanosine triphosphate (GTP)-bound form is facilitated by GEFs, which promote GDP dissociation. The inactivation of the small GTPases is mediated by GTPase-activating proteins (GAPs), which enhance GTP hydrolysis (Cherfils and Zeghouf, 2013). Critically, the activation of the GTPase cycle—activation, inactivation, removal from the membrane—is transient. Oscillations of Ca^2+^_i_ may drive parallel oscillatory association and dissociation of the Ras-GAP, Rasal (Ras-GTPase–activating-like protein), to and from the PM. Only when bound to the PM, Rasal is active and can inactivate Ras. Thus, Ras is repetitively activated and inactivated (Walker et al., 2004). In macropinocytosis, Ras activity is terminated by RasGAP, which is recruited to the cup as it closes (Veltman et al., 2016; Buckley et al., 2020). When GTPases are persistently activated, e.g., when RasGAPs are inactive like in neurofibromatosis (Bloomfield et al., 2015; Ghoshal et al., 2019) or when Ras is constitutively active like in oncogenic H-Ras mutants, macropinosome formation and maturation deviate from the normal physiological pathway. The Ras-related G protein Rap1 is a negative regulator of Ras (Zwartkruis and Bos, 1999; Nussinov et al., 2020). Rap1 is found in early and late endocytic vesicles and lysosomes (Pizon et al., 1994), and its overexpression negatively regulates macropinocytosis (Seastone et al., 1999). In *Dictyostelium discoideum*, hyperosmotic stress activates Rap1 and decreases endocytic activity due to cellular acidification (Pintsch et al., 2001), and it promotes the formation of giant vacuoles of pinocytotic origin (Yuan and Chia, 2001).

Notably, in human intestinal cells, hypotonicity increases the activity of the H-Ras–Raf1–Erk signaling pathway (Van Der Wijk et al., 1998). The clustering of Ras proteins in distinct microdomains at the PM (lipid rafts) influences Ras structure, orientation, and Ras-isoform accessibility and thus the activation states of its effectors. Accordingly, GTP-bound H-Ras may remain in a locked state and as such not able to associate with its downstream effectors (Jang et al., 2016; Nussinov et al., 2018). Activation occurs once the lipid-anchored GTPases Ras1 and H-Ras are shifted out of the lipid rafts. This happens upon thinning of the PM in combination with changes of its curvature induced by cell swelling (Cohen, 2018).

Active Rac and Ras are located at the cup wall. Rac1 activation is associated with ruffle formation, and Rac1 inactivation precedes cup closure (Buckley and King, 2017). In *Dictyostelium discoideum*, the multidomain protein, RGBARG (RCC1, RhoGEF, BAR, and RasGAP-containing protein), orchestrates Ras and Rac activity in a small membrane patch, where RGBARG is localized at the protruding rim region (Buckley et al., 2020). Oncogenic H-Ras promotes the translocation of the vacuolar H^+^-ATPase (v-ATPase) from intracellular vesicles to the PM. The accumulation of v-ATPase is necessary for the cholesterol-dependent association of Rac1 with the PM, which is a prerequisite for the stimulation of membrane ruffling and macropinocytosis. Knockdown of the v-ATPase or its inhibition suppresses macropinocytosis, while addition of cholesterol to these cells restores both Rac1 membrane localization and macropinocytosis (Ramirez et al., 2019). In addition, Ras binds and activates phosphatidylinositol 3-kinases (PI3Ks), which have a Ras-binding domain (Gupta et al., 2007; Castellano and Downward, 2011; Castellano et al., 2013). Activation of PI3Ks phosphorylates phosphatidylinositol 4,5-bisphosphate [PI(4,5)P2] to phosphatidylinositol (3,4,5)-trisphosphate [PI(3,4,5)P3] and promotes its enrichment in the PM (Rupper et al., 2001; Nussinov et al., 2020). In EGF-stimulated A431 cells, PI(4,5)P2 increases in the ruffles-forming macropinocytic cups, reaches its maximum just before macropinosome closure, and then rapidly falls as the cup closes. In contrast, PI(3,4,5)P3 increases locally at the site of macropinosome formation and peaks at the time of closure (Araki et al., 2007). The extension of PI(3,4,5)P3 patches, which can reach a diameter of several micrometers, reflects the balanced activity of PI3Ks and the lipid phosphatase, phosphatase and tensin homolog (PTEN), which dephosphorylates PI(3,4,5)P3 back to PI(4,5)P2. The kinetics of PI(4,5)P2 and PI(3,4,5)P3 are mechanistically linked to actin-remodeling during macropinocytosis (Araki et al., 1996, 2007; Worby and Dixon, 2014; Swanson and Yoshida, 2019; Nussinov et al., 2020) and to the regulation of many ion channels and transporters relevant to pinocytosis as well as CVR (Araki et al., 1996; Lang et al., 1998; Ritter et al., 2001; Furst et al., 2002; Jakab et al., 2002; Okada, 2004; Lang, 2007; Suh and Hille, 2008; Hoffmann et al., 2009; Abu Jawdeh et al., 2011; Lang and Hoffmann, 2012; Balla, 2013; Hansen, 2015; Hille et al., 2015; Kunzelmann, 2015; Jentsch, 2016; Pasantes-Morales, 2016; De Los Heros et al., 2018; Delpire and Gagnon, 2018; Konig et al., 2019; Okada et al., 2019; Centeio et al., 2020; Larsen and Hoffmann, 2020; Model et al., 2020). For cup closure, the progressive dephosphorylation of PI(3,4,5)P3 seems to be important. Among its dephosphorylation products, PI(3)P has been shown to directly activate the Ca^2+^-activated K^+^-channel, KCa3.1, at ruffles, which is necessary for closure (Maekawa et al., 2014). The significance of Ras activation, which peaks shortly after cup closure (Welliver and Swanson, 2012; Egami et al., 2014), as well as of the antagonistic behavior of PI3Ks and PTEN in the initiation and termination of cup formation is nicely documented by the observations that injection of Ras in cells induces ruffle formation, that inhibitors of PI3K inhibit cup closure in macrophages, and that deletion of PTEN in prostate cancer cells enhances macropinocytosis (Bohdanowicz and Grinstein, 2013; Levin et al., 2015; Kim et al., 2018; King and Kay, 2019).

Phosphatidylinositol (3,4,5)-trisphosphate enrichment promotes the translocation of the serine/threonine protein kinase Akt/PKB *via* binding to the pleckstrin homology (PH) domain and kinase activation by target of rapamycin complex 2 (TORC2) and 3-phosphoinositide-dependent protein kinase-1 (PDK1) (Yoshida et al., 2018; Kay et al., 2019). Among the downstream targets of Akt is the tuberous sclerosis complex 2 (TSC2), which is—together with TSC1—located on the lysosomal membrane, from which it subsequently dissociates to act as a GAP for the Ras-related small GTPase, Rheb, which in turn activates mammalian target of rapamycin complex 1 (mTORC1). In *Dictyostelium discoideum* downstream of PI(3,4,5)P3, the homologs of mammalian Akt, PkbA, and of the related glucocorticoid-regulated kinase (SGK), PkbR1, as well as their activating protein kinases, TORC2 and PdkA, increase the size of the macropinocytic patch and macropinosome (Kay et al., 2019; Williams et al., 2019). Combined inhibition of mTORC1/mTORC2 induces massive catastrophic macropinocytosis, i.e., methuosis, in cancer cells (Srivastava et al., 2019). Akt and TORC2 target SGK1, which regulates a plentitude of cell functions, including ion channels and transporters (Lang et al., 2018) and endomembrane trafficking (Zhu et al., 2015). SGK transcription is stimulated by cell shrinkage *via* p38-kinase and inhibited by cell swelling due to transcriptional stop (Waldegger et al., 1997, 2000; Lang et al., 2006, 2018). Furthermore, the isoform Akt3 controls actin-dependent macropinocytosis in macrophages by suppressing the expression of with no lysine kinase 2 (WNK2) and the activity of SGK1, while increased activity of SGK1 leads to stimulation of Cdc42-mediated macropinocytosis of lipoprotein (Ding et al., 2017). However, in macrophages stimulated by macrophage colony-stimulating factor (M-CSF), the Akt pathway is induced by this growth factor but not required for macropinosome formation (Yoshida et al., 2015).

In myoblasts, an acute decrease of PM tension leads to phospholipase D2 activation, production of phosphatidic acid, F-actin and development of PI(4,5)P2-enriched membrane ruffling, and macropinocytosis without an increase in PI(3,4,5)P3 (Loh et al., 2019).

In parallel to the lipid and protein phosphorylation cascades, the cortical actin filaments are reorganized. This is controlled by PI3K and PLC, Rac, Cdc42, Arf6, Rab5, and Pak (Swanson, 2008). In the cup region, polymerized actin is seen as a ring-like structure (Hinze and Boucrot, 2018). Active Ras and PIP(3,4,5)P3 coincidentally form patches in macropinosomes, which are surrounded by a ring of the Scar/WAVE complex, an activator of the Arp2/3 complex. Arp2/3 drives actin polymerization by filament branching, leading to the formation of dendritic F-actin, which populates the wall of the cup and forms rings of protrusive actin under the PM and the circular ruffles (Swanson, 2008; Saarikangas et al., 2010; Pollard, 2016; Buckley and King, 2017; Hinze and Boucrot, 2018; Kay et al., 2019; Williamson and Donaldson, 2019). PI(3,4,5)P3 also recruits myosin proteins to macropinocytic cups (Chen et al., 2012). The synthesis of PI(3,4,5)P3 from PI(4,5)P2 occurs simultaneously with the recruitment of Rab5 to the PM in COS-7 cells expressing oncogenic H-Ras^G12V^ (Porat-Shliom et al., 2008). Rab5 promotes macropinosome sealing and scission downstream of ruffling. To this end, Rab5-containing vesicles are recruited to circular ruffles of the PM, which requires soluble N-ethylmaleimide-sensitive-factor attachment receptor (SNARE)-dependent endomembrane fusion. This is paralleled by the disappearance of PI(4,5)P2 and accompanies macropinosome closure. The removal of PI(4,5)P2 is dependent on Rab5 through its recruitment of the inositol 5-phosphatase Inpp5b/OCRL and *via* APPL1, an adaptor protein that regulates vesicle trafficking and endosomal signaling (Maxson et al., 2021). Rab5 and RN-tre, which is a Rab5-specific GAP as well as a Rab5 effector, are also recruited to the PM. RN-tre interacts with actin as well as actinin-4, which contributes to actin bundling (Lanzetti et al., 2004). The Rab5 cycle is active on nascent macropinosomes and stabilizes the macropinosomes. Rab5 activity is increased on macropinosome tubules (Feliciano et al., 2011). In BHK-21 cells, H-Ras^G12V^ has been shown to separately activate Rab5 and Rac1 *via* distinct Ras signal transduction pathways. While Rab5 activation stimulates pinocytosis, Rac1 stimulation causes membrane ruffling but does not contribute to the stimulation of pinocytosis (Li et al., 1997).

### Intracellular Trafficking of Macropinosomes

In *Dictyostelium*, the nascent vesicles lose their actin coat within 1 min after pinching off and internalization (Lee and Knecht, 2002). In rat basophilic leukemia (RBL) cells, “*pinosomes*… *ignite a burst of actin polymerization when they are pinched off from the plasma membrane*” and “*then move into the cytosol at the tips of short-lived actin ‘comet tails,’*” which fade within 2 min after their appearance. These brief bursts of actin polymerization are thought to help move the vesicles into the cytosol (Merrifield et al., 1999). They also require recruitment of annexin-2 to nascent macropinosome membranes as an essential prerequisite for actin polymerization-dependent vesicle locomotion (Merrifield et al., 2001).

Organelle shrinkage concentrates the to-be-digested material and recycles membrane back to the PM. Macropinosomes show two routes of structural adaptations to maximize the organelle surface area and to minimize its volume: tubulation and shrinkage (Freeman and Grinstein, 2018; King and Kay, 2019; Chadwick et al., 2021b). Macropinosomes retrieve v-ATPase from fusion with other vesicles and mature toward acidic organelles that contain hydrolytic enzymes, such as proteases, nucleases, and lipases, which are required for the degeneration of macromolecules, as well as transporters facilitating the efflux of cholesterol, cystine (the disulfide form of cysteine, which is generated during protein degradation), amino acids, cobalamin, and inorganic ions (Neuhaus et al., 2002; Buckley and King, 2017; Trivedi et al., 2020).

Mobilization and maturation of macropinosomes depend on the decoration of the vesicle membrane with distinct small GTPases (Egami et al., 2014; Egami, 2016). Transient activation of ADP-ribosylation factor 6 (Arf6), a member of the Ras superfamily, by the exchange factor, EFA6, promotes PM protrusion, formation of macropinosomes, and recycling of the vesicle membrane back to the PM (Brown et al., 2001). Arf6 colocalizes and activates phosphatidylinositol 4-phosphate 5-kinase (PIP 5-kinase), which phosphorylates phosphatidylinositol 4-phosphate PI(4)P to PI(4,5)P2 (Egami et al., 2014). Subsequently, PI(4,5)P2 and Cdc42-GTP coordinate the activation of Wiskott–Aldrich syndrome protein (WASP), which promotes the actin-nucleating and actin filament-branching activity of Arp2/3 (Higgs and Pollard, 2000). The recruitment of Arf6 and its exchange factor, ARF nucleotide binding-site opener (ARNO), from cytosol to endosomal membranes relies on v-ATPase-dependent intra-endosomal acidification, thus regulating the protein-degradative pathway (Hurtado-Lorenzo et al., 2006). Persistent activation of Arf6, as seen in the GTP hydrolysis-resistant mutant Arf6^Q67L^ or by overexpression of the PIP 5-kinase, results in the accumulation of macropinosomes. Interestingly, PI(4,5)P2-enriched macropinosomes in apposition to each other fuse with one another and give rise to large vacuoles. Furthermore, entrapped PM proteins in these vacuoles are not recycled (Brown et al., 2001). The GTPase septin is involved in endosome fusion. Whereas septin downregulation decreases macropinosome fusion events as well as lysosomal delivery, septin overexpression increases delivery to lysosomes (Dolat and Spiliotis, 2016).

Early macropinosomes harbor Rab5 (Feliciano et al., 2011; Maxson et al., 2021), which in turn recruits the class III PI3K Vps34, which catalyzes the reaction from PI to PI(3)P (Christoforidis et al., 1999; Kerr and Teasdale, 2009). In macropinosomes routed toward lysosomes, Rab5 is replaced by Rab7 (Racoosin and Swanson, 1993; Kerr et al., 2006; Langemeyer et al., 2018; Morishita et al., 2019). In an analogy to an electrical safety-breaker, Del Conte-Zerial et al. (2008) compare the replacement of Rab5 by Rab7 as a “cut-out switch.” This model predicts that Rab5 drives Rab7 activation above a distinct threshold. Above the threshold, Rab7 shows a self-sustained activity and suppresses Rab5 activity *via* a negative feedback. In this model, crossing the Rab7 activation threshold excludes reactivation of Rab5 and activation of a different trafficking pathway. Thus, the Rab5 to Rab7 switch ensures a unidirectional route of cargo-loaded macropinosomes toward lysosomes. Using Förster/fluorescence resonance energy transfer (FRET) imaging, Morishita et al. (2019) describe that active Rab5 facilitates Rab7 activation until Rab7 sustains its own activity and inactivates Rab5. Furthermore, recruitment of amyotrophic lateral sclerosis 2 (ALS2) to the macropinosome coincides with Rab5 activation, and ALS2 detachment is associated with Rab5 inactivation (Morishita et al., 2019). In earlier studies using Texas red-labeled dextran macropinosomes, Racoosin and Swanson showed that Rab7-positive macropinosomes fuse with tubular lysosomes (Racoosin and Swanson, 1993). The fate of waste-containing vesicles is not known.

Dysfunction or inhibition of Vps34 or PIKfyve, which phosphorylates PI(3)P to PI(3,5)P2, leads to the formation of massive and progressively exacerbating cytoplasmic vacuolization due to loss of PI(3,5)P2. This requires active v-ATPase activity and a functional Rab5a cycle. Interestingly, the formation of the enlarged vacuoles does not require their acidification (Compton et al., 2016; Saveanu and Lotersztajn, 2016), pointing to an osmotic function of the v-ATPase. In melanoma cells, oncogenic class I PI3K elicits a hyperactive influx of macropinosomes, which is counteracted by Rab7A (Alonso-Curbelo et al., 2015). Furthermore, by stimulating RIN1, which is a Rab5 GEF, activation of H-Ras or H-Ras^G12V^ also mediates homotypic fusion of early endosomes, thus leading to endosome enlargement (Roberts et al., 2000; Tall et al., 2001).

Microtubules are associated with peripheral actin/myosin-enriched lamellae, and they are the scaffold for the unidirectional transport of macropinosomes. Critically, inhibitors of microtubule assembly, and the dynein inhibitor ciliobrevin D, decrease fluid uptake, indicating that the microtubules are involved in an early step of macropinocytosis (Williamson and Donaldson, 2019).

## Cell Volume Regulation

In general, cells respond to alterations of the osmotic equilibrium with a change of their volume due to water movement into or out of the cell. While an increase in intracellular or a decrease in extracellular osmolarity leads to cell swelling, a decrease in intracellular or an increase in extracellular osmolarity leads to cell shrinkage. Physiologically, such changes arise when cells invade anisotonic extracellular environments, e.g., the renal medulla, or following changes in intracellular osmolyte concentrations, such as during uptake or release of ions or nutrients, but also from metabolic changes, such as formation or degradation of macromolecules, e.g., proteins or glycogen. Likewise, perturbations of the double Donnan equilibrium, established by the so-called pump–leak balance (Okada, 2004; Kay, 2017; Kay and Blaustein, 2019), such as changes in intracellular pH (pH_i_) or inhibition of the Na^+^/K^+^-ATPase by cardiac glycosides or low temperature (Russo et al., 2015), will lead to alterations of CV (for review, see Lang et al., 1998; Ritter et al., 2001; Furst et al., 2002; Jakab et al., 2002; Okada, 2004, 2020; Lang, 2007; Hoffmann et al., 2009; Kunzelmann, 2015; Jentsch, 2016; Pasantes-Morales, 2016; De Los Heros et al., 2018; Delpire and Gagnon, 2018; Okada et al., 2019; Centeio et al., 2020; Larsen and Hoffmann, 2020; Model et al., 2020).

The kinetics and degree of the actual volume changes critically depend on the water permeability of the PM, which is intrinsically low but greatly enhanced by aquaporins (AQPs) (Day et al., 2014; Kitchen et al., 2015) and also by the efficiency as well as the time of onset of CVR mechanisms. If the regulatory mechanisms [i.e., regulatory volume decrease (RVD) and regulatory volume increase (RVI); see below] have high transport efficiency and/or will start to work quickly, the degree of swelling or shrinkage will deviate from a perfect osmometer-like behavior.

When the actual CV deviates from the given set point, regulatory mechanisms are spurred, aiming at readjusting the original volume. Thus, upon swelling, cells initiate a process termed RVD (cells shrink back toward their original volume), while cell shrinkage is counteracted by RVI (cells swell back toward their original volume). The mechanisms driving the regulatory water fluxes during RVD and RVI are complex and involve rapid release or uptake of ions, amino acids, sugars, or alcohols across the cell membrane but also metabolic changes such as the formation or degradation of macromolecules to pack or unpack abundant osmotically active solutes.

Acute CVR relies on distinct sets of ion channels and transporters. The Na^+^/K^+^-ATPase actively pumps K^+^ into and Na^+^ out of the cell. K^+^ permeating through K^+^ channels generates a negative PM potential Ψ_PM_ and thus creates the driving force for the cellular exit of anions such as Cl^–^ and HCO_3_^–^. Canonically, during RVD, volume-sensitive K^+^ and anion channels, KCl cotransport, or parallel activation of K^+^/H^+^ exchange and Cl^–^/HCO_3_^–^ exchange is activated, while during RVI, Na^+^/H^+^ exchangers (NHEs) and Na^+^/K^+^/2Cl^–^ cotransporters (NKCCs) in parallel to Cl^–^/HCO_3_^–^ exchange or Na^+^ channels are activated to release or take up ions and osmotically obliged water through AQPs. Thus, RVD is mainly accomplished *via* cellular exit of K^+^, Cl^–^, and HCO_3_^–^, whereas RVI is achieved by uptake of Na^+^ and Cl^–^. Frequently, RVD mechanisms are inhibited during RVI and *vice versa*.

Furthermore, shrunken cells can accumulate organic osmolytes such as amino acids, myoinositol, betaine, and taurine either by synthesis or by Na^+^-coupled uptake of sorbitol, glycerophosphorylcholine, and monomeric amino acids. These osmolytes are then released upon cell swelling. Though inhibition of the Na^+^/K^+^-ATPase leads to cell swelling at least in some cells (Alvarez-Leefmans et al., 1992), interestingly, in certain cell types, CVR can also be performed by formation and exocytosis of vesicles even when the Na^+^/K^+^-ATPase is inhibited (Russo et al., 2015).

Cell volume also greatly affects macromolecular crowding, i.e., the concentration of macromolecules (mainly proteins and nucleic acids) within the cell and hence their biological activities, which in turn has widespread consequences for cellular functions, including CVR itself (Model et al., 2020). Macromolecular crowding also induces liquid–liquid phase separation as part of the cellular osmosensing system (Ishihara et al., 2021; Watanabe et al., 2021).

As mentioned above, in myoblasts, an acute decrease of PM tension induces macropinocytosis. This was established by hypotonic swelling (stretching the PM and inducing RVD) of the cells followed by returning to isosmotic conditions (inducing cell shrinkage and PM relaxation) (Loh et al., 2019).

**FIGURE 3:**
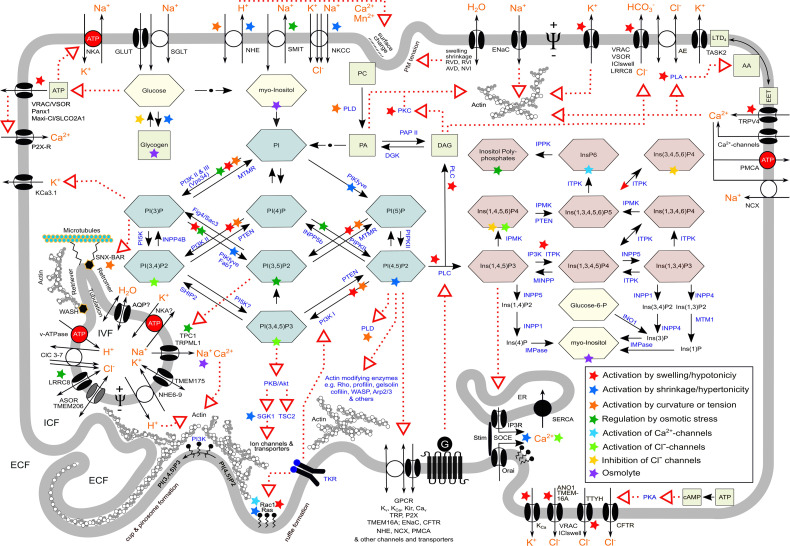
Synopsis of various aspects in macropinocytosis and ion transport in cell volume regulation and vesicular volume regulation, which are also relevant in methuosis. Red dotted arrows indicate action/s on target/s; black arrows indicate metabolic conversion; green combs, phosphoinositides; red combs, inositolphosphates; yellow combs, sugars; green squares, important metabolites; orange letters, ion(s); blue letters, enzymes; ECF, extracellular fluid; ICF, intracellular fluid; IVF, intravesicular fluid; G, heterotrimeric G protein; TKR, tyrosine kinase receptor; NKA, Na^+^/K^+^-ATPase; SOCE, store-operated Ca^2+^ entry; Ψ, transmembrane electrical potential difference. For the meaning of colored asterisks, see insert in the right lower part. For details, nomenclature, and further abbreviations, see text and list of abbreviations.

Both PIPs and IPs are involved in CVR. As shown in Figure 3, PI(4,5)P2 is metabolized to Ins(1,4,5)P3 and further to the various inositol (poly)phosphates (Hatch and York, 2010). Prominently, Ins(1,4,5)P3 binds to its receptor on internal Ca^2+^ stores like the endoplasmic reticulum (ER) and triggers the release of Ca^2+^ into the cytosol (Berridge, 2009). Given the plentitude of cellular functions governed by Ca^2+^_i_ and the numerous components of CVR dependent on it, Ins(1,4,5)P3 is crucial to it. Notably, the levels of IPs are altered in Ras-transformed cells (Fleischman et al., 1986; Maly et al., 1995; Ritter et al., 1997a). Besides, other inositol (poly)phosphates are activated or inhibited by anisotonicity and/or changes in CV (Fleischman et al., 1986; Lang et al., 1998; Jakab et al., 2002; Pesesse et al., 2004; Hoffmann et al., 2009; Wundenberg and Mayr, 2012; Lee et al., 2020).

In programmed CD, cell shrinkage is characteristic (though not universal) of apoptosis and in its initial phase accomplished by RVD-like cellular exit of ions, termed apoptotic volume decrease (AVD). In contrast, cell swelling is characteristic of necrosis and ischemic cell death/onkosis (derived from the Greek word *ó*γκ**o*ς*, i.e., tumor/swelling Von Recklinghausen, 1910; Majno and Joris, 1995; Weerasinghe and Buja, 2012), termed necrotic volume increase (NVI) (Okada et al., 2001; Orlov and Hamet, 2004; Lang and Hoffmann, 2012, 2013a,b; Orlov et al., 2013; Bortner and Cidlowski, 2014, 2020; Model, 2014; Okada, 2020), and related modes of CD such as secondary necrosis, pyroptosis, or ferroptosis (Zong and Thompson, 2006; D’Arcy, 2019; Nirmala and Lopus, 2020; Riegman et al., 2020). In methousis, not only is cell shrinkage absent but cells actually are swollen (Overmeyer et al., 2008), such as seen in cells expressing an activated form of the H-Ras^G12V^ oncoprotein (see below) (Maltese and Overmeyer, 2014; Alonso-Curbelo et al., 2015). This may be related to altered ion transport in cells expressing the H-Ras oncogene.

The set point of a given cell for regulation of its volume is not a fixed constant but may change intrinsically to adjust the CV to altered functional needs. For instance, proliferating cells have to gain volume prior to mitosis, and hence, the set point for volume regulation varies in a cell cycle-dependent manner (Lang et al., 1992a, 2007; Doroshenko et al., 2001; Chen et al., 2002; Klausen et al., 2007; Pedersen et al., 2013). Expression of H-Ras^G12V^ in NIH 3T3 fibroblasts leads to an upshift of the set point for CVR, i.e., the cells swell (Lang et al., 1992a). This is related to alterations of cellular metabolism, ion transport, and structural remodeling. Such cells proliferate independently of growth factors and have altered PI metabolism (Fleischman et al., 1986; Maly et al., 1995), stimulated Ca^2+^ influx (Maly et al., 1995; Ritter et al., 1997b), and increased intracellular pH (pH_i_) due to activation of NHE (Maly et al., 1989; Ritter et al., 1997b) and Na^+^, K^+^, 2C1^–^ cotransport (NKCC1) (Lang et al., 1992a). Furthermore, mitogens, like serum or bradykinin, cause pulsatile release of Ca^2+^ from internal stores and activation of a store-operated calcium entry (SOCE), which leads to oscillations of Ψ_PM_ *via* Ca^2+^-activated K^+^ channels, NHE activation, and actin depolymerization (Lang et al., 1992b; Dartsch et al., 1994b, 1995; Ritter et al., 1997b). In NIH 3T3 fibroblasts that do not express the H-Ras oncogene, bradykinin causes a single transient hyperpolarization, is without effect on actin stress fibers, and leads to cell shrinkage, unless actin is depolymerized (Lang et al., 1992b) (for review, see Ritter and Woll, 1996). The H-Ras-induced Ψ_PM_ oscillations are stimulated by hypertonic cell shrinkage (Ritter et al., 1993) presumably *via* fostering physical apposition and hence interaction of STIM and Orai, which accomplish SOCE (Lang et al., 2018). In line with that, SOCE has been shown to be inhibited by cell swelling (Liu et al., 2010). Furthermore, SOCE is stimulated by hypertonicity *via* NFAT5 (TonEBP) (Sahu et al., 2017) and SGK1 (Lang et al., 2018). Oncogenic K-Ras^G13*D*^ has been shown to suppress SOCE by altered expression of STIM1 in colorectal cancer cells (Huang and Rane, 1993; Pierro et al., 2018). Interestingly, fibroblastic L cells also display spontaneous oscillations of the cell membrane potential driven by oscillations of Ca^2+^_i_ and concomitant K^+^ channel activation. These oscillations are modulated by low- and high-density lipoproteins in parallel with Ca^2+^-dependent stimulation of their pinocytosis (Tsuchiya et al., 1981; Rane, 1991; Huang and Rane, 1993). Oncogenic H-Ras also activates volume-regulated anion channels (VRACs) (Schneider et al., 2008). An inverse relationship between CV and cell number has been described for T24H-Ras (H-Ras^G12V^) bearing Rat1 and M1 fibroblasts (Kunzschughart et al., 1995).

## Water, Ions, and Their Channels and Transporters in Pinocytosis

### Extracellular Ionic Composition, Osmolarity, and Extracellular pH

The ionic composition of the extracellular fluid affects pinocytosis by modulating the surface charge of the cell membrane and the cell membrane potential. Electrostatic interactions with the [normally negative (Cevc, 1990)] outer surface charge of a cell and/or the presence of positive charges in macromolecules play an important role in the induction of pinocytosis. In *Amoeba proteus*, monovalent cations induce pinocytosis in the order Cs^+^>K^+^>Na^+^>Li^+^, while divalent cations are less effective. Critically, Ca^2+^ and Mn^2+^ reduce the sensitivity of monovalent cations but are themselves without effects on pinocytosis (Stockem, 1966; Josefsson, 1968; Josefsson et al., 1975). Furthermore, the initial binding of pinocytosis inducers to the outer cell surface promotes displacement of surface-associated Ca^2+^ ions and induces changes in various membrane parameters, such as increased membrane conductance and decreased Ψ_PM_ along with an increase in plasmalemma hydration. Although pinocytosis in the amoeba can be induced by a great number of different solutes, all of them characteristically possess net positive charges that interact with negative surface charges. Hence, increasing extracellular Na^+^ displaces most of the exchangeable surface-associated Ca^2+^ and, therefore, induces pinocytosis. In the yolk sac, cationic macromolecules lead to pinocytosis (Lloyd, 1990). In human corneal epithelial cells, fibroblasts, and human umbilical vein endothelial cells (HUVECs), the uptake of an antibody–drug conjugate by macropinocytosis is facilitated by the presence of positive charges or hydrophobic residues on the surface of the macromolecule (Zhao et al., 2018). In contrast, in mammalian macrophages, anionic molecules are better inducers of pinocytosis than neutral or cationic ones (Cohn and Parks, 1967). The uptake of positively charged nanoparticles in colon carcinoma Caco-2 cells is reduced by inhibition of macropinocytosis with 5-(*N*-ethyl-*N*-isopropyl)-amiloride (EIPA) and by cholesterol depletion of the PM, whereas these inhibitors have no effect on negatively charged systems (Bannunah et al., 2014). Modulation of the charge for intracellular delivery carriers aims at increasing the efficiency of macropinocytosis of cellular entry for therapeutic nucleic acids to tumor cells (Desai et al., 2019).

Anisotonicity modulates endocytosis in a diversity of cell types. In rat Kupffer cells, hypoosmotic and hyperosmotic conditions have been shown to stimulate and inhibit, respectively, phagocytosis (Warskulat et al., 1996), and in microglial BV-2 cells, preconditioning with hypotonic or hypertonic medium attenuates microsphere uptake (Harl et al., 2013). Hypertonic medium has been shown to disrupt the interaction of caveolae with endosomes. Increased phosphorylation due to phosphatase inhibition induces removal of caveolae from the PM. In the presence of hypertonic medium, this is followed by their redistribution to the center of the cell close to the microtubule-organizing center (Parton et al., 1994).

Only a few studies have addressed the action of anisotonicity on pinocytosis. In *Dictyostelium*, hyperosmotic conditions lead to a decrease in endocytic activity that can be attributed to cellular acidification (Pintsch et al., 2001), and it can foster the formation of giant vacuoles of pinocytotic origin, which appear to have a function in cellular osmoregulation (see above) (Yuan and Chia, 2001). In murine bone marrow-derived macrophages, hypotonic cell swelling stimulates phagocytosis and pinocytosis, both of which appear to require ClC-3 chloride channels. In this study, the ion channels have on the one hand been suggested to act as volume-activated anion channels in the PM and, on the other hand, shown to support acidification the endosomal compartment (Yan et al., 2014). Hypertonicity inhibits pinocytosis in rat hepatocytes (Synnes et al., 1999). In mouse L929 fibroblasts, cellular uptake of horseradish peroxidase is inhibited in hypertonic sucrose medium, which is reversed to stimulated uptake in the presence 10% PEG 1000 in the medium (Okada and Rechsteiner, 1982; Hughey et al., 2007). Similarly, in murine embryonic fibroblasts, cellular uptake of native proteins *via* macropinocytosis in hypertonic conditions is stimulated by alkali metal ions (Na^+^, Rb^+^, K^+^, Li^+^) but not by hypertonicity created by addition of sugars or sugar alcohols. This finding points to the contribution of surface charge in macropinocytosis. Also, hypotonic stress has been demonstrated to greatly enhance receptor-independent retroviral transduction efficiency in NIH 3T3 fibroblasts *via* stimulated intensive endocytosis (Lee and Peng, 2009).

An interesting concept explaining how extracellular pH elicits such effects is that protonation of the cell surface produces local charge asymmetries across the cell membrane, which induce inward bendings of the lipid bilayer, thus favoring vesicle formation and uptake of macromolecules (Ben-Dov and Korenstein, 2012).

In dendritic cells and bone marrow-derived macrophages, extracellular acidosis improves uptake and presentation of antigens by stimulation of macropinocytosis (Vermeulen et al., 2004; Martínez et al., 2007). This effect is related to acid-sensing ion channels (ASICs) and can be inhibited by their blocker amiloride (Kong et al., 2013). As macropinocytosis depends on phospholipase C (Amyere et al., 2002; Yoshida et al., 2015), its activation by proton-sensing G protein-coupled receptors such as ovarian cancer G protein-coupled receptor 1 (OGR1/GPR68), G protein-coupled receptor 4 (GPR4), T-cell death-associated gene 8 (TDAG8/GPR65), and GPR132/G2A (Seuwen et al., 2006; Alexander et al., 2017; Insel et al., 2020) may also explain the observed stimulatory effect of extracellular acidosis on pinocytosis. Notably, in HEK293 cells expressing mutated wild-type OGR1 or mutated OGR1^L74P^, receptor internalization, Ca^2+^_i_ mobilization, and morphological changes observed upon activation of OGR1 by H^+^ or Ni^2+^ are severely compromised. This missense mutation of the H^+^ ion-sensing receptor has been found to cause familial amelogenesis imperfecta (Sato et al., 2020). Experiments with HEK293 cells transfected with active OGR1 receptor or a mutant lacking five histidine residues (H5Phe-OGR1) unraveled that receptor activation by H^+^ stimulates NHE- and v-ATPase activity only in OGR1- but not in H5Phe-OGR1-transfected cells. Furthermore, the known OGR1 inhibitors Zn^2+^ and Cu^2+^ reduce the stimulatory effect (Mohebbi et al., 2012). Given the importance of NHE1 and the v-ATPase for pinocytosis, it is tempting to speculate that OGR1 is a regulator thereof.

By contrast, in pancreatic acinar cells, low extracellular pH selectively impairs apical endocytosis. This is seen in mice lacking cystic fibrosis transmembrane conductance regulator (CFTR), which normally couples endocytosis at the apical PM to HCO_3_^–^ secretion into the ductal lumen by normally rendering it alkaline. The acidic luminal fluid and impaired endocytosis due to lack of CFTR can be restored by alkalizing it *in vitro* (Freedman et al., 2001).

### Ion Fluxes Drive Gel–Sol Transitions of the Cortical Actin Rim

Reorganization of the submembranous cytoskeleton is an essential step in pinocytosis. The cortical actin network is a major determinant of cell stiffness, and a correlation between stiffness of the actin network and the activity of endocytosis has been demonstrated (Planade et al., 2019). Extracellular K^+^ and Na^+^ antagonistically modulate the gel-to-sol transition of the cortical actin cytoskeleton beneath the PM (for review, see Oberleithner et al., 2009; Callies et al., 2011; Oberleithner and De Wardener, 2011; Warnock et al., 2014). Elevations of extracellular Na^+^ and K^+^ concentration stiffen and soften, respectively, the submembranous actin cytoskeleton of endothelial cells within minutes (Oberleithner et al., 2009). The Na^+^-dependent stiffening is mediated by an aldosterone-induced upregulation and activation of epithelial Na^+^ channels (ENaCs) and, presumably, by downregulation of the endothelial nitric oxide synthase (eNOS) activity. Conversely, inhibition of ENaCs upregulates nitric oxide [NO; formerly termed endothelium-derived relaxing factor (EDRF)] production. Depolarizing Ψ_PM_ by increasing extracellular K^+^ concentration, by blocking K^+^ channels with Ba^2+^, and by decreasing extracellular Cl^–^ concentration decreases the mechanical stiffness of endothelial cells (Callies et al., 2011). Thus, modulation of the sol–gel transition of the actin cytoskeleton is thought to be due to the G-actin-dependent activation of eNOS (Fels et al., 2010).

It has to be mentioned that hypotonic swelling of endothelial cells was shown to cause stiffening of the PM due to an increase in cellular hydrostatic pressure rather than to disruption of the submembranous actin network. In this study, a change in membrane tension was not observed upon osmotic swelling, and depolymerization of F-actin did not abrogate swelling-induced stiffening of the PM (Ayee et al., 2018).

### Cell Volume, Nitric Oxide, and the Cytoskeleton May Act Together in Regulating Endocytosis

In endothelial cells, a reciprocal regulatory relationship between eNOS and caveolin-1 (Cav-1) has been found, whereby Cav-1 and eNOS regulate the function of each other. On the one hand, Cav-1 stabilizes eNOS expression and regulates its activity, and eNOS-derived NO promotes caveolae-mediated endocytosis of albumin and insulin; on the other hand, a sustained NO production and persistent S-nitrosylation of Cav-1 lead to its ubiquitination and degradation (Chen et al., 2018).

Besides eNOS, also an association of inducible NO synthase (iNOS) with the submembranous actin cytoskeleton and intracytoplasmic vesicles in lipopolysaccharide (LPS)- and interferon gamma (INFγ)-stimulated macrophages and other cells was shown (Webb et al., 2001; Su et al., 2005).

Nitric oxide is a regulator of endocytosis, phagocytosis, and vesicle trafficking (Weinberg, 1998; Chen et al., 2018). For example, NO downregulates endocytosis in rat liver endothelial cells (Martinez et al., 1996) but promotes caveolae-mediated endocytosis as mentioned above (Chen et al., 2018). Macrophages stimulated by the thymic peptide thyαl display enhanced NO levels as well as increased pinocytosis (Shrivastava et al., 2004), as do amphotericin B-stimulated microglial cells (Kawabe et al., 2017). Conversely, mouse peritoneal macrophages stimulated with activin A display both reduced NO and pinocytosis (Zhou et al., 2009). Also, macropinocytosis of drugs may promote enhancement of iNOS expression, as exemplified for the chemotherapeutic nab-paclitaxel, which synergizes to this end with INFγ (Cullis et al., 2017).

Recently, it has been shown that NO regulates endocytic vesicle budding by *S*-nitrosylation of dynamin—a GTPase that regulates vesicle budding from the PM—and increases its enzymatic activity in response to NO (Wang et al., 2006). Dynamin is bound by NOS trafficking inducer (NOSTRIN), an eNOS-interacting adaptor protein, which forms a complex with Cav-1 and eNOS, and colocalizes with WASP and actin to promote its polymerization. It thus regulates caveolar endocytosis and eNOS internalization (Schilling et al., 2006; Su, 2014).

Nitric oxide/EDRF is also released in response to changes in extracellular osmolarity, which also greatly affect the cortical actin organization. This is also relevant in apoptosis, where NO metabolism, actin organization, and CV-regulatory ion transport are linked together (Bortner, 2005).

Nitric oxide release in response to hypertonicity occurs in vessels and endothelial cells (Steenbergen and Bohlen, 1993; Vacca et al., 1996; Zani and Bohlen, 2005). Hypertonicity downregulates eNOS in human aortic endothelial cells (HAECs), an effect that is mediated by activation of AQP1 and NHE-1, and which involves PKCβ-mediated intracellular signaling (Madonna et al., 2010). In insulin-treated HAECs, hypertonicity, established by high glucose or mannitol, downregulates the PI3K/Akt/mTOR/eNOS pathway and impairs their ability to respond to insulin. This may contribute to insulin resistance. The mechanism involves AQP1 and the transcription factor Ton/EBP (NFAT5) for osmosensing, and the effect can be reversed by silencing the transcription of these proteins (Madonna et al., 2020).

Hypotonic swelling has been shown to promote the release of NO and reactive nitrogen oxide species in various cells (Kimura et al., 2000; Haussinger and Schliess, 2005; Takeda-Nakazawa et al., 2007; Kruczek et al., 2009; Gonano et al., 2014) and to augment LPS-triggered iNOS expression in RAW 264.7 macrophages (Warskulat et al., 1998). Recently, it has been shown that in HUVECs, VRAC/SWELL1/LRRC8A mediates endothelial cell alignment *via* stretch-dependent Akt-eNOS signaling and formation of a signaling complex made up by Grb2, Cav-1, and eNOS (Alghanem et al., 2021).

While osmotic shrinkage in general is associated with an increase in actin polymerization, cell swelling leads to its depolymerization, though both with exceptions (Hoffmann et al., 2009).

Hypertonic shrinkage fosters the translocation of the actin-binding protein cortactin to the cortical actin net, where it interacts with the Arp2/3 complex, WASP, dynamin, and myosin light-chain kinase (for review, see Pedersen et al., 2001; Hoffmann et al., 2009). Cortactin stabilizes microfilament assembly at the cell periphery, is recruited to PM ruffles, and participates in macropinocytosis (Mettlen et al., 2006). In human-induced pluripotent stem cells, hyperosmolarity, created by high glucose or mannitol, upregulates AQP1 and induces cytoskeletal remodeling with increased ratios of F-actin to G-actin, effects that could be by reversed siRNA-mediated inhibition of AQP1 expression (Madonna et al., 2014).

Cell swelling can lead to reorganization of intermediate filaments (Dartsch et al., 1994a; Li J. et al., 2019). In Cos-7 fibroblast-like cells, hypotonicity causes a rapid calcium/calpain-dependent cleavage of the intermediate filament vimentin, whereby hypotonicity leads to the generation of IP_3_ by hydrolysis of PI(4,5)P2 and Ca^2+^ release from the ER (Pan et al., 2019). Vimentin in turn is involved in the regulation of vesicular trafficking (Margiotta and Bucci, 2016) and was also shown to be modulated by NO (Sripathi et al., 2012).

### Na^+^/H^+^ Exchangers and Pinosome Formation

A distinctive hallmark to distinguish clathrin-dependent endocytosis from clathrin-independent macropinocytosis is the sensitivity of the latter to inhibitors of NHEs such as amiloride, EIPA, or HOE-694 (West et al., 1989; Koivusalo et al., 2010; Canton, 2018; Lin et al., 2020). Besides NHEs, amiloride also directly inhibits ion channels of the epithelial sodium channel/degenerin family, such as ENaC and ASIC1 (Kellenberger and Schild, 2002; Qadri et al., 2010) and growth factor receptor tyrosine kinase activity (Davis and Czech, 1985). Moreover, amiloride is a weak permeant base that can accumulate in acidic vesicles, thus, eventually dissipating the H^+^-gradient driving cation exchange, and thereby inhibiting pinocytosis (Dubinsky and Frizzell, 1983; Bakker-Grunwald et al., 1986). Of note, EIPA does not inhibit a newly described form of CD, which is induced by fenretinide, which is similar to methuosis characterized by hyperstimulated macropinocytosis and massive vacuolization (Brack et al., 2020).

Na^+^/H^+^ exchangers, which are members of the solute carrier (SLC) 9 family, are electroneutral transporters that exchange Na^+^ for H^+^ across membranes (Pedersen and Counillon, 2019). The NHE1 isoform is expressed almost ubiquitously and regulates cellular and organelle pH, motility, phagocytosis, proliferation, as well as cell survival and CD (Orlowski and Grinstein, 2011; Pedersen and Counillon, 2019). Cell shrinkage is a strong stimulator of NHE1 activity and serves for RVI (see above) (Ritter et al., 2001; Baumgartner et al., 2004; Orlowski and Grinstein, 2011; Pedersen and Counillon, 2019). NHE1 is activated by a drop in pH_i_, a broad diversity of PM receptors, such as tyrosine kinase receptors, G-protein-coupled receptors or integrin receptors, and it is regulated by second messengers, asymmetric membrane tensions, and phosphoinositides, such as PI(4,5)P2 (Orlowski and Grinstein, 2011; Pang et al., 2012; Pedersen and Counillon, 2019). In proximal tubular cells, NHE1 is activated by PI(4,5)P2 and inhibited by PI(3,4,5)P3 (Abu Jawdeh et al., 2011; Pang et al., 2012). NHE1-borne changes in pH_i_ may also be confined to distinct subcellular domains or compartments. Such subcellular compartments are the lammelipodia formed during cell spreading and migration, pseudopodia in phagocytosis, ruffles and the cup wall formation in macropinocytosis. In all of these processes, NHE1 is also involved in cytoskeletal rearrangement (Schwab and Stock, 2014; Pedersen and Counillon, 2019). NHE1 is connected to actin *via* ezrin/radixin/moesin (ERM) family of actin-binding proteins. Migrating cells undergo cell polarity-dependent subcellular volume changes. Repetitive cycles of protrusion at the leading edge (lamellipodium) are followed by retraction of the cell’s rear, i.e., the trailing edge. Waves of local water movement across the PM locally increase and decrease CV during the protrusion and retraction of the lamellipodium, respectively. Hence, cell migration requires an alternating cycle of subcellular RVI at the leading edge and RVD at the trailing edge of the cell. Most of the ion transporters known to participate in CVR are also involved in the regulation of cell migration. As part of the RVI transporters, NHE1 is confined to the lamellipodium, where it also serves for local extracellular acidification (Lang et al., 1998; Schneider et al., 2000; Loitto et al., 2002, 2009; Jakab and Ritter, 2006; Hoffmann et al., 2009; Schwab et al., 2012; Stock et al., 2013). This is also given in breast cancer cell invadopodia, where NHE1 is allosterically regulated by NaV1.5 Na^+^ channels (Brisson et al., 2012, 2013). In T47D human breast cancer cells, NHE1 colocalizes with Akt and ERK in prolactin-induced ruffles (Pedraz-Cuesta et al., 2016).

Although inhibition of macropinocytosis by NHE inhibitors is a fingerprint feature of macropinocytosis, the molecular mechanisms how NHEs contribute to macropinocytosis are barely known. Interestingly, activation of the small GTPases, Rac1/Cdc42, and consequently actin polymerization and, thus, ruffle formation are sensitive to submembranous pH (pH_sm_). Submembranous acidification due to inhibition of NHE1 by amiloride prevents Rac1/Cdc42 activation and suppresses actin polymerization (Koivusalo et al., 2010). Moreover, NHE1 inhibition favors the accumulation of cytosolic H^+^, which neutralizes the negative charges on the inner leaflet of the PM. Thus the interaction of the negatively charged headgroups of phosphoinositides and the polybasic motifs in Rac and Cdc42 as well as the SCAR/WAVE complex and WASP is hampered. This also explains the inhibitory effect of NHE1 blockers on macropinocytosis (Marques et al., 2017). Therefore, amiloride might not directly inhibit macropinocytosis but cause submembranous accumulation of metabolically generated H^+^ in thin lamellipodium-like membrane extensions by inhibition of H^+^ efflux. Given the small volume of these structures, only a few H^+^ will change pH_sm_ and, consequently, the state of actin polymerization. However, in HeLa cells, oncogenic Ras-induced macropinocytosis does not require a decrease in pH_sm_ (Ramirez et al., 2019).

Notably, in mice bearing xenograft tumors derived from oncogenic K-Ras bearing MIA PaCa-2 cells, intratumoral macropinocytosis and tumor growth are suppressed by treatment of the animals with EIPA, which is, however, effective only in tumors with high but not low macropinocytic activity (Commisso et al., 2013; Recouvreux and Commisso, 2017).

The ionophoric NHE monensin induces vesicular swelling and the formation of giant multivesicular bodies (MVBs) (Stein and Sussman, 1986; Savina et al., 2003) and release of exosomes, which can be inhibited by the NHE inhibitor dimethyl amiloride (DMA) (Chalmin et al., 2010; Pironti et al., 2015). Moreover, deletion of the *Saccharomyces cerevisiae* NHE, Nhx1, disrupts the fusogenicity of the MVB in a manner dependent on pH and monovalent cation gradients (Karim and Brett, 2018).

### The Ion Composition in Macropinosomes and Endolysosomal Vesicles

After pinching off, the nascent macropinosome has entrapped extracellular fluid that equals the extracellular fluid in the intimate vicinity of its formation site. Traveling along the endolysosomal pathway, its ionic composition is modified to render the vesicular volume, shape, and fluid suitable for processing its cargo, as shown in Table 1 (Scott and Gruenberg, 2011; Freeman and Grinstein, 2018; Chadwick et al., 2021b).

### Water Permeability, Aquaporins, and Osmolarity

The permeabilities of the PM and vesicular membranes for water play crucial roles in pinocytosis. The importance of PM AQPs for macropinocytosis is seen in dendritic cells where they are needed as essential elements of a CV control mechanism necessary to concentrate the macrosolutes and present antigens (De Baey and Lanzavecchia, 2000; Hara-Chikuma et al., 2011; Kitchen et al., 2015).

Macropinosomes shrink along their cellular route by moving ions and osmotically obliged water out of its lumen into the cytosol (Freeman and Grinstein, 2018; Chadwick et al., 2021b). Despite the dramatic decrease in pinosome volume, the water permeation pathway is not characterized yet. Studies on osmotic water permeability (Pf) indicate that Pf values higher than 0.01 cm/s indicate water flux through channels (Verkman et al., 1996), Pf values in the range from 0.003 to 0.005 cm/s designate membranes lacking water channels, and Pf values between 0.0001 and 0.005 cm/s characterize pure phospholipid bilayers (Fettiplace and Haydon, 1980; Echevarria and Verkman, 1992; Olbrich et al., 2000). These values could be used to evaluate the presence or absence of water channels in endosomes.

Clathrin-coated vesicles from bovine brain that lack water channels have a low Pf of ∼0.001 cm/s and retain this value after stripping off the coat. Vesicles prepared from bovine renal cortex and inner medulla revealed two populations: one containing water channels with high (0.02 cm/s) and one lacking them with low values compared to brain-derived vesicles (Verkman et al., 1989). Vasopressin-induced endosomes of rat kidney papilla and toad bladder endocytic vesicles have Pf values of 0.03 and >0.1 cm/s, respectively, whereas isolated toad bladder granules have a Pf value as low as 0.0005 cm/s (Verkman and Masur, 1988; Verkman et al., 1988; Shi and Verkman, 1989; Verkman, 1989).

Only a few studies have investigated Pf of pinosomes and lysosomes. In J774 macrophages, the Pf of the PM is in the range of ∼0.004–0.009 cm/s (Fischbarg et al., 1989; Ye et al., 1989; Echevarria and Verkman, 1992). The water permeability of the macropinosome membrane of J774.A1 cells increases non-linearly from ∼0.001 cm/s 3 min after formation to ∼0.005 cm/s after 25 min of formation (Chaurra-Arboleda, 2009). The lysosomal Pf of CHO-K1 cells is in the same range (Chaurra et al., 2011). In J774.A1 cells, Pf shows pH dependency. It decreases from ∼0.007 to 0.0009 cm/s following lysosomal alkalinization with NH_4_Cl (pH_lys_ 6.5–6.8) and to 0.0001 cm/s after inhibition of the v-ATPase (pH_lys_ ∼7.0). These values are similar to those in early macropinosomes, which have a pH of ∼6.7 (Chaurra-Arboleda, 2009). Taken together, the low Pf values of J774 macrophages indicate the absence of AQPs in the endolysosomal compartment.

Despite these specific observations, the role of AQPs in the endolysosomal compartment is poorly investigated, while their importance in volume regulation of secretory vesicles is well documented (Cho and Jena, 2006; Sugiya et al., 2008; Jena, 2020). In toad urinary bladder endosomes, AQP-TB has been shown to be present (Siner et al., 1996), and incorporation of PM-derived AQP2 into endosomes is well known for AVP-stimulated renal collecting duct principal cells (Shi and Verkman, 1989; Verkman, 2005). In astrocytes, AQP4-laden vesicles may fuse with the PM upon cell swelling (Potokar et al., 2013; Vardjan et al., 2015). AQP6 was shown to be present in intracellular vesicles of acid-secreting intercalated cells of the renal collecting duct where it colocalizes with the H^+^-ATPase and serves also as an anion channel (Yasui et al., 1999; King et al., 2004). Lack of AQP11 causes defective endosomal pH regulation, as seen in mice devoid of AQP11. This is accompanied by the appearance of huge vacuoles in the renal proximal tubules and polycystic kidneys (Ishibashi et al., 2009).

An example for the function of organellar AQPs in VVR and CVR is evident in *Trypanosoma cruzi*, as briefly described below.

Alternatively to AQPs, water permeation through transporters and ion channels has been described for glucose transporters (Loike et al., 1993; Fischbarg and Vera, 1995), NKCC1, KCC, SGLT1, and CFTR (Hamann et al., 2010; Zeuthen, 2010; Zeuthen and Macaulay, 2012; Huang et al., 2017). Eventually, these transporters or ion channels could be used in pinosomes for water efflux as well.

By whatever way, as upon hypertonic cell shrinkage also vesicles rapidly shrink, it can be deduced that the intrinsic water permeability is sufficiently high to allow for timely vesicular volume changes during resolution of macropinosomes (Freeman and Grinstein, 2018). Using fluorescence enhancement of Lucifer yellow dextran by deuterated water, Li et al. (2020) have recently demonstrated that lysosomes rapidly swell in response to a hypoosmotic challenge, indicating that there is substantial water influx into the lumen of lysosomes soon after water penetration across the PM, again indicating that the intrinsic water permeability of these organelles is high.

### Vesicular pH Regulation and Ions in Pinosome Maturation

The pH of the ingested fluid of nascent pinosomes resembles that of the extracellular fluid, which is under physiological conditions (7.4). During trafficking, vesicular pH (pH_ves_) gradually decreases to the value prevailing in lysosomes, i.e., 4.5–5.0. pH_ves_ regulates enzyme activities and enables oxidation reactions, the release of internalized receptors from their ligands and their recycling back to the PM, movement and assembly of organellar surface coat proteins, vesicle maturation, as well as membrane fusion processes (Demaurex, 2002; Ohgaki et al., 2011).

#### Vacuolar ATPase

The acidification is primarily achieved by insertion of the v-ATPase. The v-ATPase is an evolutionarily highly conserved primary active H^+^ transporter. As already outlined, it can be found both in the PM and in various intracellular organelles. Structurally, it comprises a multiprotein complex that forms two distinct domains, i.e., the pore-forming transmembrane V_O_ domain and the cytosolic V_1_ domain. The latter hydrolyzes ATP to drive H^+^ movement. This creates the electrochemical H^+^ gradient across the membrane, thereby driving secondary active transport processes, which act together for proper adjustment of pH_ves_ and vesicular ion and osmolyte composition. In particular, parallel to H^+^ pumping, ion channels and transporters move Cl^–^, H^+^, and other cations to establish an electrical shut aiming for electroneutrality of the net charge transfer. Otherwise, the acidification would be limited by the transmembrane potential Ψ_ves_, which is set up by the v-ATPase itself (Demaurex, 2002; Steinberg et al., 2010; Koivusalo et al., 2011; Xiong and Zhu, 2016; Freeman and Grinstein, 2018; Jentsch and Pusch, 2018; Sterea et al., 2018; Li P. et al., 2019; Freeman et al., 2020; Chadwick et al., 2021a). pH_ves_ is stabilized by the buffering capacity of the vesicle content (∼60 mM/pH at pH 4.5–5) (Weisz, 2003; Steinberg et al., 2010).

In addition, the v-ATPase also serves as a protein interaction hub. For the proper sorting and targeting of the vesicles, small GTPases are recruited to their membranes in an acidification-dependent manner. The v-ATPase itself is able to associate with some of them and with other regulatory proteins. Thus, the v-ATPase seems not only to serve for creating the acidic pH_ves_ but also to sense it and to transmit this information to its cytoplasmic domain, thus enabling trafficking molecules to bind and perform their targeting functions. The v-ATPase is also involved in signaling pathways for the regulation of macropinocytosis, as outlined throughout this review. Its dysfunction may play critical roles in various diseases including diabetes, cancer, neurodegeneration, osteopetrosis, skin disorders, or renal tubular acidosis and other pathologies. Furthermore, it is also essential for viral entry into cells (for reviews on v-ATPase, see Nishi and Forgac, 2002; Platt et al., 2012; Maxson and Grinstein, 2014; Rappaport et al., 2016; Kissing et al., 2018; Futai et al., 2019; Collins and Forgac, 2020; Song Q. et al., 2020; Vasanthakumar and Rubinstein, 2020; Chadwick et al., 2021a, b; Eaton et al., 2021).

#### Cl^–^ Ions

Within 1 min after internalization, the luminal Cl^–^ concentration in endosomes/macropinosomes drops from ∼120–150 mM to ∼20 mM (Sonawane et al., 2002). This decrease is insensitive to Cl^–^ channel inhibition and can be attributed to Cl^–^ expulsion by an interior negative Donnan potential (Ohshima and Ohki, 1985; Sonawane and Verkman, 2003; Hryciw et al., 2012). During maturation, the vesicular Cl^–^ concentration increases again up to ∼130 mM in lysosomes (Saha et al., 2015). The Cl^–^ accumulation of late endosomes can be suppressed by inhibition of the v-ATPase and restored by the K^+^ ionophore valinomycin. Also, replacement of Cl^–^ by gluconate and Cl^–^ channel inhibition slow endosomal acidification. Thus, Cl^–^ is an important counter ion accompanying endosomal acidification (Sonawane and Verkman, 2003) (for review, see Faundez and Hartzell, 2004; Stauber and Jentsch, 2013). The accumulation of lysosomal Cl^–^ appears to be important for the adjustment of the lysosomal volume and the activity of proteases. Reduced lysosomal Cl^–^ concentrations may lead to lysosomal storage diseases, e.g., Gaucher’s disease (OMIM entries 230800, 230900, 231000) or Nieman–Pick’s disease (OMIN entries 257200, 607616, 257220, 607625) (Cigić and Pain, 1999; Platt et al., 2012, 2018; Stauber and Jentsch, 2013; Rappaport et al., 2016; Chakraborty et al., 2017; Astaburuaga et al., 2019).

The Cl^–^ channels and transporters expressed in intracellular organelles include the ClC family members ClC-3 through 7, chloride intracellular channels (CLICs), CFTR, AQP6, transmembrane proteins (TMEM)16C–G/anoctamin (ANO) 3–7, bestrophin-1, Golgi pH regulator (GPHR) (reviewed in Stauber et al., 2012; Stauber and Jentsch, 2013), Tweety homolog 1 (Ttyh1) proteins (Wiernasz et al., 2014), LRRC8 (Li et al., 2020), and TMEM206 (Osei-Owusu et al., 2021).

The ClC-3 to 7 transporters are confined to distinct endolysosomal compartments with partially overlapping appearance (Jentsch and Pusch, 2018). They are electrogenic outwardly rectifying 2Cl^–^/H^+^ exchangers working in parallel with the v-ATPase. As per exchange cycle, two Cl^–^ enter and one H^+^ leaves the vesicle, three negative charges accumulate in the vesicle. To maintain electroneutrality, three H^+^ are pumped in, one of which leaves the vesicle again *via* the 2Cl^–^/H^+^ exchanger, thus leading to a net uptake of two H^+^ (Guzman et al., 2013; Jentsch and Pusch, 2018). This proposed mechanism allows a more efficient H^+^ and Cl^–^ accumulation, as it generates a more inside-negative Ψ_ves_ than an ohmic Cl^–^ conductance could do. Yet, this mechanism may be restricted to ClC-5 (Zifarelli, 2015). However, Ψ_ves_ is generally thought to be negative (i.e., positive in the lumen) due to the rheogenic v-ATPase. The reason for this discrepancy is still elusive (Weinert et al., 2010) (reviewed in Jentsch, 2007; Stauber et al., 2012; Stauber and Jentsch, 2013; Jentsch and Pusch, 2018). Dysfunctional ClC exchangers may lead to a broad variety of symptoms and disorders including neurodegeneration and other neuropathies, proteinuria and kidney stones, osteopetrosis, albinism, and lysosomal storage diseases (Jentsch and Pusch, 2018; Nicoli et al., 2019; Schwappach, 2020; Bose et al., 2021). Overexpression of ClC-3 or gain-of-function mutations of ClC-6 or ClC-7/Ostm1 leads to swelling of late endosomes and lysosomes (Li et al., 2002; Nicoli et al., 2019; Polovitskaya et al., 2020). In B-cell non-Hodgkin lymphoma cells, the PIKFyfe inhibitor apilimod leads to CD by formation of giant vacuoles and disruption of endolysosomal function, an effect that requires functional ClC-7/Ostm1 transporters (Gayle et al., 2017). In Hela and NIH3T3 cells, a short natural ClC3 splice variant (Clc3s) has been shown to lead to the formation of large vacuoles (Wu et al., 2016), and in Chinese hamster ovary CHO-K1 or human hepatoma Huh-7 cells, the volume of such vesicles is governed by their Cl^–^ concentration (Li et al., 2002).

Endothelial cells lacking CLIC4 display defective endothelial cell tubulogenesis and impaired acidification of large intracellular vesicles, while lysosomes are unaffected (Ulmasov et al., 2009).

Previous work showed that cell swelling leads to alkalinization of acidic cellular vesicles, regardless whether the cells are swollen by hypotonicity, isoosmotically with high-K^+^ solutions, inhibition of K^+^ channels, or concentrative uptake of solutes such as amino acids. At least in liver cells, swelling-induced alkalinization occurs rather in the pre-lysosomal than lysosomal compartments. The increased pH_ves_ affects proteolysis, trafficking of cell membrane proteins, and antigen presentation (Busch et al., 1994, 1996, 1997; Völkl et al., 1994). The mechanisms leading to swelling-induced rise of pH_ves_ are still elusive. Interestingly, overexpression of a naturally occurring C-terminally truncated splice variant of mouse bestrophin-3, Best3V2, leads to, besides swelling, alkalinization of lysosomes (Wu et al., 2016). Manipulation of the intracellular Cl^–^ concentration leads to alterations of lysosomal volume due to the high ClC-3-endowed Cl^–^ permeability. Accordingly, a decrease in intracellular Cl^–^ concentration during RVD following cell swelling could alter pH_ves_. In H-Ras oncogene-expressing fibroblasts, which have a higher CV (see above), the swelling-induced vesicular alkalinization is less pronounced compared to cells not expressing the oncogene. Hence, in H-Ras-expressing cells, any effect of the swelling-induced vesicle alkalinization on cell function may be altered (Busch et al., 1997). Notably, murine and human fibroblasts expressing oncogenic K-Ras also display significant alkalinization of lysosomes (Jiang et al., 1990).

In several cell types, the fusion of late endosomes to lysosomes is prevented by isotonic K^+^ buffers as a consequence of an increased permeability of cells to K^+^ and concomitant cell swelling. This inhibition is selective for late endosomes, since other endosome fusion events, such as homotypic fusion of early or late endosomes or fusion of recycling endosomes with the PM, are not affected. Cell swelling is regarded to be causative for this effect (Ward et al., 1990). As cell swelling and lack of fusion of endosomes to lysosomes are also hallmarks of methuosis (see below and Figure 2), it is tempting to speculate that cell swelling in methuosis might not only be a passive consequence of swollen vacuoles but also be a cause of it.

A link between CVR and vacuolar pH regulation is also established by the proteins MLC1 and GliaLCAM, which are defective in megalencephalic leukoencephalopathy with subcortical cysts. This rare congenital disease is characterized by macrocephaly, ataxia, seizures, degeneration of motor functions, and cognitive decline, morphologically by chronic white matter edema and subcortical cysts, and on the ultrastructural level by intra-myelinic vacuole formation and enlarged intracellular vacuoles (Van Der Knaap et al., 2012). The protein MLC1 is involved in astrocytic CVR. It may be a volume-sensitive ion channel itself, but it is also part of a macromolecular complex composed of the Na^+^/K^+^-ATPase, Kir4.1 K^+^-channels, AQP4, syntrophin, and caveolin-1, as well as volume-sensitive TRPV4 cation channels, which mediate cellular Ca^2+^ influx upon cell swelling. In addition, MLC1 also influences CLC-2 chloride channels as well as the volume-sensitive anion channel(s)/current(s) (VRACs) and therefore also RVD. It has been shown that knockdown of LRRC8 annihilates the potentiating effect of MLC1 on VRAC currents *via* modulation of the phosphorylation state of the channel subunit LRRC8C (Elorza-Vidal et al., 2018). GlialCAM serves as an escort protein for MLC1 and CLC-2, and it is necessary for its proper activation by cell swelling. MLC1 is expressed in early and recycling endosomes, which they use to travel to the PM during hyposmotic stress (Capdevila-Nortes et al., 2013). Importantly, MLC1 also interacts with the v-ATPase, and it is involved in regulating early endosomal pH (Lanciotti et al., 2012). Defective MLC1 may result in impaired recycling and retention of TRPV4 channels in the cytoplasmic perinuclear area and thus disturbed swelling-induced cellular Ca^2+^ influx (Lanciotti et al., 2012) such as seen in monocyte-derived macrophages from MLC patients (Ridder et al., 2011; Petrini et al., 2013; Brignone et al., 2014, 2015; Elorza-Vidal et al., 2018). Furthermore, CLC-2 knockout mice exhibit myelin vacuolization, which is thought to arise from dysregulation of extracellular ion concentrations (Stauber et al., 2012). Moreover, ClC-2 channels are functionally regulated by SGKs by inhibiting the ubiquitin ligase Nedd4-2, which in turn results in reduced clearance of ClC-2 protein from the PM (Palmada et al., 2004). As outlined above, SGK1 is involved in the regulation of macropinocytosis, and hence, both ClC-2 and SGKs may be tight together in the regulation of macropinocytosis.

The acid-activated outwardly rectifying chloride channel/current (ASOR; also termed proton-activated, outwardly rectifying anion current, PAC, PAORAC; or *I*_*Cl,H*_) could be a candidate to serve as electrical shut for endolysosomal acidification. This anion channel or its core component is made up by TMEM206 proteins, which form a trimeric channel that is architecturally related to ENaCs/degenerin channels and ASICs (Ullrich et al., 2019; Ruan et al., 2020; Deng et al., 2021). TMEM206 has been shown to interact with Akt (Zhao et al., 2019) and to contribute to acid-induced CD (Osei-Owusu et al., 2020). The ASOR current is characterized by activation at pH values < 5.0, strong outward rectification, activation at positive transmembrane potentials, and sensitivity to typical Cl^–^ channel blockers (Sato-Numata et al., 2013, 2017; Kittl et al., 2019, 2020; Ullrich et al., 2019). In contrast to CLCs, which are inhibited by extracellular/luminal acidic pH (Scheel et al., 2005; Jentsch, 2007; Jentsch and Pusch, 2018), ASOR is activated by it. Accordingly, endosomal ASOR and CLCs could regulate Ψ_ves_ and vesicular ion concentrations. TMEM206 is mainly localized in the PM, but variable cytoplasmic labeling has been documented (Ullrich et al., 2019). Recently, it has been shown that TMEM206 indeed traffics from the PM to endosomes. Its deletion annihilates the endosomal Cl^–^ conductance, raises the luminal Cl^–^ concentration, lowers pH_ves_, and increases transferrin receptor-mediated endocytosis. Moreover, its overexpression generates a large endosomal Cl^–^ current with properties resembling the endogenous conductance and reduces endosomal acidification as well as transferrin uptake. Thus, endosomal TMEM206 appears to function as a sensor for low pH and may prevent hyperacidification by releasing Cl^–^ from the lumen (Osei-Owusu et al., 2021). Clearly, the role of TMEM206/ASOR/PAORAC in the regulation for Ψ_ves_ and pH_ves_ warrants further investigations. Likewise, its role in VVR needs to be investigated.

LRRC8A-E/SWELL1 proteins are essential components of the volume-regulated outwardly rectifying Cl^–^ current (VRAC, VSOR, VSOAC, ICl_swell_, ICl_vol_) elicited in most cells during cell swelling (Qiu et al., 2014; Voss et al., 2014; Jentsch, 2016; Jentsch et al., 2016; Okada, 2020; Bertelli et al., 2021), and they are involved in a broad variety of cellular functions (Chen et al., 2019). LRRC8A-E/SWELL1 also regulate the PI3K-Akt, Erk1/2, mTOR signaling cascade (Kumar et al., 2020; Alghanem et al., 2021). Recently, Li et al. (2020) demonstrated the expression of LRRC8 family members on lysosomal membranes and their ability to build functional lysosomal VRACs (Lyso-VRACs). Patch clamp investigation of these vacuoles revealed that Lyso-VRACs are activated by ionic strength and permeable for anions, like Cl^–^, NO_3_^–^, HCO_3_^–^, acetate, aspartate, or glutamate. They are inhibited by known VRAC blockers. Early endosome membranes lack Lyso-VRACs. Hypoosmotic swelling promotes the formation of large cytoplasmic vacuoles containing markers for late endosomes and lysosomes with a diameter of >2 μm, which require functional Lyso-VRACs. Loss of VRAC channels enhances necrotic CD triggered by sustained hypoosmotic, hypoxic, and hypothermic stress. Furthermore, the authors demonstrated that endolysosome-derived giant vacuoles are subject to exocytosis. This releases PM tension and simultaneously reduces CV. Thus, by acting as water store-and-release compartments, the swelling-induced giant vesicles have been compared to the “*cell’s ‘bladder,’ sequestering intracellular excess water through vacuolation and then expelling the potentially toxic level of water through exocytosis, which also relieves the plasma membrane tension stress”* (Li et al., 2020).

AQP6 is activated by low pH and acts as Cl^–^ channel in acid-secreting α-intercalated cells in renal collecting ducts. There it is found in endosomes where it colocalizes along with ClC-5 and the v-ATPase. However, ClC-5 is inhibited at acidic pH, whereas the anion conductance of AQP6 is turned on. Hence, ClC-5 may rather operate when acidification of the vesicles starts, whereas AQP6 may take over this role as their pH drops. Furthermore, AQP6, though its water permeability is low, may mediate vesicle swelling and membrane fusion during exocytosis or other cellular processes (Yasui et al., 1999; Hazama et al., 2002; Kitchen et al., 2015).

#### HCO_3_^–^ Ions

The role of HCO_3_^–^ in pinocytosis has not met much attention yet. Carbonic anhydrase (CA) catalyzes the dissociation of HCO_3_^–^ to generate H^+^ of CO_2_. CA isoforms are present at the PM, in the cytosol, and in lysosomes (Rikihisa, 1985; Reibring et al., 2014). There they associate with numerous acid–base transporters such as anion exchangers 1-3 (AE1-3), sodium bicarbonate cotransporters NBCe1 and NBCn1, and NHE1 (Becker et al., 2014). Recently, Sedlyarov et al. (2018) found that electroneutral NBCn1 (NBC3, SLC4A7) is essential for phagosome acidification in macrophages. NBCn1 resides in the PM, and lack of it leads to cytoplasmic acidification, which in turn hampers phagosome maturation and impairs bacterial killing. If membrane-associated CA is internalized during the formation of the pinosomes, it will catalyze the dissociation of HCO_3_^–^ to generate CO_2_, which in turn will rapidly equilibrate across the vesicular membrane. Notably, in global CVR, VRAC may actually work more effectively in driving RVD by passing HCO_3_^–^ than Cl^–^, as the former can be virtually unlimitedly replenished from CO_2_ in the presence of CA, whereas Cl^–^ can only exit the cell as long as the Ψ_PM_ is more negative than the equilibrium potential for this ion (Völkl and Lang, 1988; Ritter et al., 1991). In line with these considerations, the VRAC channels made up of LRRC8A with B through E subunits are permeable to HCO_3_^–^ (Gaitan-Penas et al., 2016). Hence, Lyso-VRAC may provide a vesicular HCO_3_^–^-conductive pathway as well.

#### Na^+^ Ions

Among the internalized ion transporters, the Na^+^/K^+^-ATPase may limit endosomal acidification. Its rheogenic Na^+^ transport contributes to the Ψ_vesicle_ of endosomes, thereby opposing H^+^ transport. This is, however, only evident in early but not in late endosomes (Fuchs et al., 1989). In line with this, it has been shown in Swiss 3T3 fibroblasts that inhibition of the Na^+^/K^+^-ATPase by ouabain strongly enhances the acidification of early but not late endosomes or lysosomes. Ouabain has also been shown to produce stronger endosomal acidification and parallel Cl^–^ accumulation in transferrin-labeled early and recycling endosomes of J774 cells (Sonawane and Verkman, 2003). Ouabain exerts its effect by acting on the interior side of the vesicles rather than on the PM (Zen et al., 1992). In contrast, in early rat liver endosomes, the Na^+^/K^+^-ATPase does not regulate acidification (Anbari et al., 1994).

Whether internalized PM retrieved NHE1 contributes to pinosomal acidification remains to be determined. According to the prevailing Na^+^ gradient between the lumen and the cytosol, NHEs should serve to acidify nascent macropinosomes. NHE1 does, however, not play a direct role in phagosome acidification due to the rapidly dissipating Na^+^ gradient between the phagosome and the cytosol and the absence of the Na^+^/K^+^-ATPase to maintain such a gradient. Instead, phagosomal acidification is established by the v-ATPase (Hackam et al., 1997, 1999). By analogy, this also may hold true for macropinosomes.

The human orthologs of yeast NHX1 are the endosomal Na^+^(K^+^)/H^+^ exchangers NHE-6 and NHE-9. NHE-6 is present in early and NHE-9 in late recycling vesicles. NHE-6 is also inserted into the PM upon vesicular recycling. NHE-6 and NHE-9 bind to the adaptor protein for activated PKC, receptor for activated C kinase (RACK1), which interacts with metabolic enzymes, kinase receptors, and ion transporters and contributes to the maintenance of pH_ves_ by regulating the distribution of the transporters between endosomes and the PM. They are involved in clathrin-dependent endocytosis by alkalinizing early endocytic vesicles. Endosomes of NHE-6-deficient neurons appear to be strongly acidic. Such a hyperacidification occurs upon hypoxia-induced mobilization of NHE-6 to the PM (Lucien et al., 2017). In mouse, loss of NHE-6 causes endolysosomal storage disease with accumulation of gangliosides and unesterified cholesterol in late endosomes and lysosomes of neurons of selective brain regions. In humans, mutations of NHE-6, NHE-7, and NHE-9 cause neurological syndromes (Ilie et al., 2014, 2016; Kondapalli et al., 2014; Schwede et al., 2014). NHE-5 may contribute to organellar pH regulation and regulate cell surface expression of the receptor tyrosine kinase MET and the EGF receptor (Fan et al., 2016). NHE-7 is found in the *trans*-Golgi network and in endosomes and also interacts with RACK1. Interestingly, NHE-7 mediates an acidification of intracellular vesicles that adds to that set up by the v-ATPase and that accelerates endocytosis (Milosavljevic et al., 2014). NHE-8 is found in the mid- to *trans*-Golgi compartment and MVBs. It regulates late endosomal morphology and function. In HeLa-M cells, NHE-8 silencing results in perturbation of MVB protein sorting, disrupted endosomal protein trafficking, and perinuclear clustering of endosomes and lysosomes (Lawrence et al., 2010). In the kidney, it also localizes to the apical PM and regions of coated pits. NHA-2 appears to localize to multiple compartments. It is expressed in endosomes of pancreatic β cells and synaptic-like microvesicles and participates in clathrin-dependent but not -independent endocytosis (Fuster and Alexander, 2014). In lysosomes, NHEs are absent (Nakamura et al., 2005) (for review, see Donowitz et al., 2013; Pedersen and Counillon, 2019).

Na^+^/H^+^ exchangers could fulfill a dual role in pH_ves_ regulation. In endosomes, NHEs face the acidic organellar interior and the high K^+^ concentration of the cytosol and act as H^+^/K^+^ exchangers to alkalinize vesicles. Once recycled through the PM, they face the high Na^+^ concentration within the nascent vesicle. As Na^+^ is then following its gradient into the cytosol, H^+^ is transported into the vesicular lumen, thus acidifying it (Pedersen and Counillon, 2019).

#### K^+^ Ions

As mentioned above, extracellular and intracellular K^+^ greatly affects endocytosis and vesicle trafficking. After pinching off, the pinosomes may retain the channel equipment of the PM, and they may contribute to the initial changes in the ionic composition of the intra-pinosomal fluid. Given the normally high K^+^ conductance of the PM and the prevailing electrochemical driving force for K^+^, this is expected to be in favor of setting up a negative Ψ_ves_ in the nascent pinosome, which in turn would initially drive its early ionic movements.

Following endosome formation, the intraluminal K^+^ concentration changes from ∼5 mM to values in lysosomes ranging from 2 to 60 mM in a manner depending on pH_lysosome_ (Steinberg et al., 2010; Sterea et al., 2018). In maturing endosomes, the accumulation of K^+^ has been shown to be dependent on cholesterol, as its depletion impairs this process (Charlton et al., 2019).

The K^+^ channel KCa3.1 is activated *via* PI(3)P and a putative regulatory subunit that is required for Ca^2+^ gating. In addition, KCa3.1 interacts with the phosphatase myotubularin R6 (MTMR6), which dephosphorylates PI(3)P, thereby inactivating the ion channel (Srivastava et al., 2005, 2006). Using coelomocytes of *Caenorhabditis elegans*, Maekawa et al. (2014) showed that the sequential dephosphorylation of phosphoinositides [in the order PI(3,4,5)P3, PI(3,4)P2, PI(3)P, PI] by phosphoinositide phosphatases, which are activated after ruffle formation, is related to the activation of KCa3.1. Before induction of macropinocytosis with EGF, KCa3.1 is enriched in “intracellular punctate structures” but not in the PM. Following EGF treatment, KCa3.1 is recruited to F-actin-positive membrane ruffles. Interestingly, exposure of coelomocytes to TRAM-34, a selective inhibitor of KCa3.1 (Panyi et al., 2006), or using a mutant KCa3.1, which is not activated by PI(3)P, impairs cellular dextran uptake. TRAM-34 treatment does not inhibit ruffle formation or actin polymerization. Interestingly, supplementation by sucrose to cells treated with TRAM-34 restores macropinocytosis, indicating that KCa3.1 contributes to macropinocytosis at least partially by regulating local osmolarity *via* a decrease of the intracellular K^+^ concentration. The authors suggest that KCa3.1 is involved in the closure of ruffles and that amiloride and TRAM-34 might be used to dissect macropinosome formation pharmacologically—amiloride inhibits the formation and TRAM-34 the closure of the ruffle (Maekawa et al., 2014).

Endosomal and lysosomal K^+^ concentration and Ψ_vesicle_ are also regulated by the selective K^+^ channel TMEM175, which in turn regulates organellar functions including fusions with each other. *Via* this channel, vesicular K^+^ concentration and Ψ_vesicle_ are hence also influenced by the cytosolic K^+^ concentration. The “resting” Ψ_lysosome_ is, however, also largely determined by the vesicular H^+^ permeability. It is predicted to be in the range of −18 mV at pH 5.0 and +12 mV at pH 4.5 when assuming a cytosolic K^+^ concentration of 140 mM (Cang et al., 2014, 2015).

Furthermore, large-conductance Ca^2+^-activated K^+^ channels (BK channels) are found in lysosomes. They are physically and functionally coupled to TRPML1 channels and facilitate release of Ca^2+^ ions, which activate further BK channels. This hyperpolarizes Ψ_lysosome_ and thus further facilitates Ca^2+^ release. BK overexpression has been shown to rescue abnormal lysosomal storage in cells from patients with the lysosomal storage disease Niemann–Pick’s disease (Cao et al., 2015).

#### Ca^2+^ Ions

Ca^2+^ ions are indispensable regulators of pinocytosis and endolysosomal functions. They induce macropinocytosis *via* F-actin depolymerization (Kabayama et al., 2009) and establish compensating exocytosis of large endosomes in parallel to ongoing macropinocytosis, thereby preventing cellular volume overload (Falcone et al., 2006). Furthermore, they regulate lysosomal fusion events and condensation of the luminal content (Xu and Ren, 2015).

Intravesicular H^+^ and Ca^2+^ concentrations are interdependently tied together. In 3T3 Swiss fibroblasts, after uptake of 2 mM extracellular Ca^2+^, the endosomal concentration rapidly drops to ∼30 μM within 3 min and further on to ∼3 μM after 20 min. This is proportionally paralleled by a drop of pH_vesicle_ from 7.4 to 7.0 and 5.7 at the same time points. The loss of vesicular Ca^2+^ can be prevented if the acidification is inhibited by blocking the v-ATPase. By reducing the external Ca^2+^ to 200 μM, the acidification is completely suppressed. Hence, the acidification can occur only when the initial Ca^2+^ concentration in the endosomes is high (Gerasimenko et al., 1998; Petersen et al., 2020).

Lysosomal Ca^2+^ homeostasis involves a putative Ca^2+^/H^+^ exchanger and Ca^2+^ pumps. The relationship between luminal Ca^2+^ and H^+^ is suggested to be as follows: the lower the pH_lysosome_, the higher the Ca^2+^ concentration (Morgan et al., 2011). In macrophages, the lysosomal Ca^2+^ concentration is ∼500 μM and dependent on extracellular and cytosolic Ca^2+^ concentration as well as on pH_lysosome_. Alkalizing pH_lumen_ reversibly decreases Ca^2+^ by shifting it into the cytosol. However, in contrast to fibroblasts, alterations of extracellular or lysosomal Ca^2+^ do not alter pH_lysosome_ in macrophages (Christensen et al., 2002; Sterea et al., 2018). This model has been challenged by the finding that lysosomal stores are rather refilled pH-independently with Ca^2+^ *via* the ER in an Ins(1,4,5)P3 receptor-dependent process and *via* involvement of an unknown Ca^2+^ transport mechanism to move Ca^2+^ into the lysosome (Garrity et al., 2016).

The Ca^2+^ permeability of the endolysosomal membranes is set up by several cation channels. Among them are the transient receptor potential channel family members TPRML1, 2, and 3 (mucolipins), two-pore channels TPC1, 2, and 3, and voltage-gated Ca^2+^ channels (Cheng et al., 2010; Abe and Puertollano, 2011; Grimm et al., 2012; Xiong and Zhu, 2016; Clement et al., 2020; Freeman et al., 2020; Jin et al., 2020).

TRP channel of mucolipin subfamilies mainly localize to lysosomes and endosomes but are also found in the PM. The channels are activated by PI(3,5)P2. TRPML1 is inhibited by PI(4,5)P2, which is abundantly present in the PM. Hence, if residing there, it is likely to be functionally suppressed (Sun et al., 2015; Venkatachalam et al., 2015).

TRPML1 is encoded by the Mcoln1 gene and expressed in late endosomes and lysosomes. It is responsible for the enlarged vacuole formation and the lysosomal storage disease mucolipidosis IV if mutated to loss of function (OMIM entry #252650) (Lloyd-Evans et al., 2008; Grimm et al., 2012). The channel is permeable to Ca^2+^, Na^+^, K^+^, Fe^2+^, and Mg^2+^, and activated by PI(3,5)P2, but inhibited by PI(4,5)P2 and sphingomyelin. TRPML1 acts as a lysosomal pH regulator as it senses the pH_lysosome_ and initiates the release of H^+^. Being activated at low pH, it allows for Ca^2+^ influx (Li et al., 2017). Accordingly, lysosomal acidification is hindered when PI(3,5)P3 generation is suppressed by inhibition or lack of PIKfyve and rescued by overexpression of TRPML1 or raising lysosomal Ca^2+^. However, the vacuole formation observed in PIKfyve-deficient cells is not rescued by Ca^2+^ or overexpressed TRPML1 (Cheng et al., 2010; Isobe et al., 2019).

PIKfyve converts PI(3)P into PI(3,5)P2 in the endocytic pathway and the enzyme Fig4 dephosphorylates it back to educt. In yeast, hyperosmotic stress leads to the rapid transient synthesis of PI(3,5)P2 *via* activation of PI(3)P kinase (Dove et al., 1997; Duex et al., 2006) and the TRPML1 ortholog Yvc1. Lack of PI(3,5)P2 synthesis leads to severe swelling of the endolysosomal compartment due to concentrating K^+^ up to ∼85 mM. This effect is relieved by inactivating mutations of the vacuolar monovalent cation/H^+^-antiporter Vnx1 or v-ATPase or by activating mutations of Yvc1. This identifies PI(3,5)P3 and the ion transporters regulated by it as crucial osmoregulators (Dove et al., 1997; Wilson et al., 2018). In contrast to yeast, hyperosmotic stress decreases and hypoosmotic treatment enhances PI(3,5)P2 production in monkey Cos-7 cells (Dove et al., 1997). PI(3,5)P2 regulates vacuole size in part *via* TRPML1 channels (Krishna et al., 2016). Hypertonicity also activates PIKfyve and its upstream regulator hVac14 in differentiated 3T3-L1 adipocytes, but not in their undifferentiated precursor cells (Sbrissa and Shisheva, 2005). In dendritic cells, TRPML1 releases lysosomal Ca^2+^ upon bacterial sensing, which activates the actin-based motor protein myosin II for directional migration. In addition, it induces the activation of the transcription factor EB (TFEB), which translocates to the nucleus to maintain TRPML1 expression. This TRPML1–TFEB axis results from the downregulation of macropinocytosis after bacterial sensing (Bretou et al., 2017). Moreover, TRPML1 is a lysosomal sensor of reactive oxygen species (ROS) (Zhang et al., 2016), which are known, on the one hand, to be generated during cell swelling (Gorg et al., 2013) and, on the other hand, to activate VRACs (Lambert, 2003; Varela et al., 2004; Browe and Baumgarten, 2006; Lambert et al., 2015). Such a link between ROS, endosomal function, and CVR may be mediated by ClC-3-dependent endosomal ROS production and isosmotic activation of VRAC (Matsuda et al., 2010).

Activating H-Ras mutations—known to drive macropinocytosis—elevates TRPML1 expression. In H-Ras-driven cancer cells, the channel is needed to restore PM cholesterol, which gets internalized during endocytosis. Inhibition or lack of TRPML1 causes false localization of cholesterol from the PM to endolysosomes and loss of oncogenic H-Ras from the cell surface (Jung and Venkatachalam, 2019). Hence, cells expressing oncogenic H-Ras are vulnerable to inhibition of TRPML1 (Jung and Venkatachalam, 2019; Jung et al., 2019).

TRPML2 is an osmo/mechanosensitive endolysosomal channel conductive to Ca^2+^, Na^+^, K^+^, and Fe^2+^ and also activated by PI(3,5)P2. It is involved in Arf6-regulated recycling pathway and in endolysosomal membrane trafficking by means of its sensitivity to hypotonicity. In late endosomes, TRPML2 is activated by free cytosolic ADP-ribose and eventually by nicotinic acid adenine dinucleotide phosphate (NAADP) (Dong et al., 2010; Viet et al., 2019). Mutations in TRPML2 may induce CD due to Ca^2+^ overload (Sun et al., 2015; Sterea et al., 2018; Chen et al., 2020a).

TRPML3 localizes to both early endosomes and endolysosomes, is Ca^2+^ permeable, activated by PI(3,5)P2, and inhibited by PI(4,5)P2, Na^+^, as well as acidic pH (Dong et al., 2010).

The two-pore channels (TPCs) are voltage-gated cation channels that function as homodimers. TPC1 localizes predominantly to the proximal endosomal system, while TPC2 is found mainly on late endosomes and lysosomes, where they act as Na^+^ and Ca^2+^ release channels. They are activated by PI(3,5)P2 (rather than NAADP) (Wang et al., 2012; Cang et al., 2013; She et al., 2019; Jin et al., 2020) and play important roles for vesicular fusion and endosomal trafficking, autophagy, nutrient sensing, protein processing, and macropinocytic virus entry (Wang et al., 2012; Bellono and Oancea, 2014; Sakurai et al., 2015; Xiong and Zhu, 2016; Castonguay et al., 2017). Inhibition or loss of TPC channels critically reduces cellular entry of severe acute respiratory syndrome coronavirus 2 (SARS-CoV-2) (Ou et al., 2020) and Ebola virus (Sakurai et al., 2015; Petersen et al., 2020). TPC1 activity is high at alkaline pH_vesicle_ and open over a wide voltage range but low at acidic pH_vesicle_. Also, low intracellular ATP concentration increases TPC1 opening. This leads to Na^+^ efflux and thus facilitates the v-ATPase and luminal acidification (Cang et al., 2014). TPC2 channels are important in organelle fusion and pH regulation. They mediate the rapid decrease of vesicular Ca^2+^ after uptake (Petersen et al., 2020). TPC2 inhibition or loss suppresses virus uptake (Sakurai et al., 2015; Petersen et al., 2020), increases the risk to develop non-alcoholic steatohepatitis (NASH) and fatty liver disease (NAFLD); Grimm et al., 2014, p. 707]. TPC2 is also involved in pigmentation (Bellono and Oancea, 2014; Lin et al., 2015).

Lysosomes seem also to possess voltage-gated calcium channel, which might serve to release Ca^2+^ upon depolarization of Ψ_vesicle_ and which are required for lysosomal fusion with endosomes and autophagosomes (Tian et al., 2015).

## Vesicular Volume Regulation

Macropinosome shrinkage or swelling is accomplished by osmotically driven exchange of water between the vesicle lumen and the cytosol (VVR) (Freeman and Grinstein, 2018; Chen et al., 2020b; King and Smythe, 2020; Chadwick et al., 2021b), in adjunction with global CVR (see above). The direction of ion flux—efflux or influx—is determined by the transmembrane ion gradients, the levels of Ψ_PM_ and Ψ_vesicle_, and the reversal potentials of the respective ions. The composition of the fluid in a nascent macropinosome initially equals the fluid in the intimate extracellular vicinity of its emergence and may be identical to the extracellular fluid. This creates a transmembrane gradient across the membrane of the macropinosome and the cytosol similar to that across the PM. Macropinosome shrinkage is therefore largely mediated by Na^+^ flux across the vesicle membrane. Recent studies demonstrated that Na^+^ efflux down its gradient from the pinosome to the cytosol is controlled by the non-selective TRPML cation channels and TPC Na^+^ channels (Laplante et al., 2002; Freeman et al., 2020; Chadwick et al., 2021a, b; Saric and Freeman, 2021).

In addition to Na^+^, also Cl^–^ permeates through the macropinosomal membrane (Freeman and Grinstein, 2018). As described above, vesicular LRRC8 channels/LysoVRACs may play a role for the flux of anions across the vesicular membrane, and their importance in VVR has been recognized (Li et al., 2020; Osei-Owusu et al., 2021). A role for TMEM206/ASOR/PAORAC seems feasible, and an eventual functional coupling of vesicular VSOR and ASOR channels/currents, as described to occur in the PM (Nobles et al., 2004; Lambert and Oberwinkler, 2005; Kittl et al., 2019, 2020), awaits future investigation.

The efflux of Na^+^ and Cl^–^ is coupled to cotransporters such as the electroneutral cation–Cl^–^ cotransporters (SLC12A family members; carrying K^+^), NHEs (SLC9A family members; carrying H^+^), and anion exchangers (AE; SLC4A family members; carrying HCO_3_^–^) (Freeman and Grinstein, 2018). Moreover, HCO_3_^–^ forms water and CO_2_, which exits the macropinosome as described above. The net result of these ion fluxes leads to osmotically driven vesicular water exit and shrinkage in parallel to H^+^ ion accumulation, thus generating small acidic late endosomes, in which the to-be-digested substances are highly concentrated (for review, see Freeman and Grinstein, 2018; Chadwick et al., 2021b).

Accordingly, macropinosome shrinkage is prevented if Na^+^ or Cl^–^ is replaced by a non-permeant cation or anion. Importantly, the osmotically driven vesicle shrinkage is necessary for the vesiculation, tubulation, and scission processes, as it confers—by creating membrane wrinkles—the appropriate curvature necessary to recruit and stabilize the protein complexes required for proper sorting and recycling of the vesicles and their cargo, e.g., anchor sites for microtubule-associated motors, branched actin generation, or cargo recognition proteins. The latter are composed of the sorting nexins (SNXs), which contain a Bin-amphiphysin-Rvs (BAR) domain. BAR domains are able to electrostatically interact with phospholipids of appropriately bent concave membrane stretches. Also, proteins of the ESCRT complexes take advantage of the slack membrane to induce inwardly budding intraluminal vesicles (ILVs) and further on MVBs. Thus, the vesicular shrinkage creates organelles with high surface-to-volume ratios, relieves the hydrostatic tension of their membranes, and is hence critical for their resolution (for review, see Freeman and Grinstein, 2018; Chen et al., 2020b; Saric and Freeman, 2020, 2021; Chadwick et al., 2021a, b).

As pointed out above, the role of and function of AQPs in VVR is still elusive. A clue about their usage may come from observations in *T. cruzi* epimastigotes. They respond to cell swelling with trafficking and fusion of acidocalcisomes, which contain osmolytes and the AQP, TcAQP1, to the so-called bladder of the contractile vacuole complex, thereby loading their content to it. This enables the bladder to take up and store excess cellular water, whereby it swells by the aid of TcAQP1. Subsequently, the water is expelled to the extracellular environment, thus driving RVD (reviewed in Docampo et al., 2011, 2013). The basic mechanistic principle of this mechanism—soak up, store, and release excess intracellular water to/from intracellular organelles—is somehow recapitulated in Cos1 cells in response to a hypotonicity as described above (Li et al., 2020). It will be interesting to find out whether this principle of linking CVR and VVR is a more general one and if it might function in other cells as well.

## Methuosis

Regulated CD in response to perturbation of the extracellular or intracellular milieu is an essential hallmark of multicellular organisms and is mediated mainly by apoptotic and necrotic phenotypes (Galluzzi et al., 2018). Methuosis is a non-apoptotic CD phenotype characterized by large and lucent vacuoles, which are limited by a single membrane as well as by cell swelling and, finally, rupture of the PM (Maltese and Overmeyer, 2014). Currently, methuosis is seen as a dysfunctional pinocytosis. Although Lewis first described macropinocytosis already in 1937 (Lewis, 1937), the fatal consequences for the cell by “drinking too much” have been described by Overmeyer et al. (2008) several decades later. While macropinocytosis is associated with vesicle shrinkage during processing of the vesicle content, methuosis reflects the abnormal growth of pinosomes. Despite that cell vacuolization is a common feature in many diseases, such as the lysosomal storage disease Gaucher’s disease or in microglia in Alzheimer’s disease, it is still uncertain whether methuosis plays a critical role in the etiology of these diseases (Kruth et al., 2005; Rappaport et al., 2016). There is also evidence that methuosis may be an eventual CD modality outcome of cell senescence (Adjemian et al., 2020).

H-Ras is required for macropinocytosis, and oncogenic mutants of a Ras allele are associated with methuosis. Chi et al. (1999) tested the assumption that the persistent activation of an oncogenic mutant of a Ras allele induces CD on those malignant tumors, which in general do not show an expression of Ras alleles. They found that transfection of oncogenic H-Ras (Ras^G12V^) in human malignant glioma cells and human gastric cancer cell lines causes CD associated with intense vacuolization (Chi et al., 1999). These authors concluded that H-Ras expression triggers a type-2 CD, meaning that, in this condition, autophagy leads to CD. Overmeyer et al. (2008) replicated, confirmed, and extended these findings, but they excluded a type-2 form of CD. As in the study of Chi et al. (1999), they used a human glioblastoma cell line and induced the expression of Ras. Within a few days, Ras-expressing cells revealed lucent cytoplasmic vacuoles in phase-contrast microscopy, which did contain neither cell organelles nor cytoplasmic components when analyzed using electron microscopy. Furthermore, electron microscopy revealed that the vacuoles are limited by a single and not by a double membrane, as would be distinctive for autophagy. Fluid-phase traces, like Lucifer yellow or dextran-Alexa Fluor 488, accumulate in large vacuoles, whereas transferrin-Alexa Fluor 594 labels small endosomes, which contain—in contrast to pinosomes—transferrin receptors. Furthermore, the small endosomes are decorated with clathrin, whereas large vacuoles do not contain a clathrin coat. In contrast to functional pinocytosis, large vacuoles are not acidic. Finally, the cells detach from the surface and swell, and the PM ruptures. Because of the similarities with pinocytosis, but the fatal consequences of fluid uptake, the authors suggest that this is a dysfunctional pinocytosis, which leads to a distinct form of CD. Accordingly, Overmeyer et al. (2008) named this form of CD methuosis, derived from the Greek word *methuo*, meaning “to drink to intoxication.”

In normal macropinocytosis, nascent macropinosomes dynamically engage with cytoskeletal elements and either are tagged for recycling or finally fuse with lysosomes. With methuosis, however, vesicles fuse with each other to form large vacuoles, leading to the presumption that methuosis is a dysfunctional pinocytosis (Maltese and Overmeyer, 2014). In molecular–biological terms, methuosis shows its relationship to macropinocytosis by its dependence on H-Ras. Despite having the late endosome markers, LAMP1 and Rab7, vacuoles are not acidic, as demonstrated by their failure to accumulate acridine orange and LysoTracker. Interestingly, inhibition of v-ATPase by bafilomycin A1 also inhibits vacuolization (Maltese and Overmeyer, 2014). An important aspect in macropinocytosis is shrinkage of the vesicles *via* osmoregulatory processes (see above). Ras and phosphoinositides are required for the activation of a variety of ion channels, including those involved in vesicle shrinkage and CVR (see above) (Ritter and Woll, 1996). Assuming that formation of large vacuoles is not only due to fusion of pinosomes but also related to organelle swelling, an imbalance in the signaling cascade from Ras to ion channels could play a key role in methuosis. In line with the crucial role of NHE1 in macropinocytosis is the observation that methuosis induced by an ursolic acid-derived small molecule, compound 17, has been shown to be prevented by EIPA (Sun et al., 2017). As outlined above, H-Ras^G12V^-expressing cells experience to shift of CVR toward higher volumes and also the intracellular vesicles have more alkaline pH. Inducers of methuosis and formation of extremely enlarged vacuoles are given in Table 2.

**TABLE 2:** Inducers of methuosis, cell death associated with aberrant vacuolization, and formation of strongly enlarged vacuoles.

**Inducers of methuosis, methuosis-like vacuolization, formation of giant vacuoles**	**Cell type/organism**	**Mode of action**	**References**
Activating H-Ras mutations	U251 and U343 human GB cells, MKN-1, TMK-1 gastric cancer cells	Activation of Rac1; inactivation of Arf6	Kaul et al., 2007; Overmeyer et al., 2008; Bhanot et al., 2010; Chi et al., 1999*
Activated Rac1	U251 human GB cells	Ras–Rac-dependent pathway	Overmeyer et al., 2008
Activating K-ras mutations	Human lung adenocarcinoma, HCK1 cervical keratinocytes; HeLa cervical cancer cells, A549 lung cancer cells	Rac1 activation	Unni et al., 2015; Dendo et al., 2018
Ionizing radiation	K-Ras^G13R^-bearing murine uterine cervix cancer (MUCC) cells	Iron-dependent CD assumed	Adjemian et al., 2020
Activating EGFR mutations	Human lung adenocarcinomas	MAPK signaling pathway	Unni et al., 2015
NGF	Daoy medulloblastoma cells	TrkA-dependent casein kinase 1 activation	Li et al., 2010
CD99 (mAB-triggered activation)	Ewing sarcoma cells	IGF-1R/Ras/Rac1 signaling	Manara et al., 2016
PFK158	Malignant pleural mesothelioma cells	Inhibition of PFKFB3	Sarkar Bhattacharya et al., 2019
OSI-027; PP242	RD, RH30 and RMS rhabdomyosarcoma cells; HeLa cervical cancer cells, MCF7 breast cancer cells; A549 lung cancer cells; A431 epidermoid cells; HaCaT and Ker-CT keratinocytes	mTORC1/mTORC2 inhibition	Srivastava et al., 2019
AS1411, G-rich quadruplex-forming oligodeoxynucleotide	DU145, MDA-MB-468, A549, LNCaP prostate cancer cells	AS1411 binding to nucleolin; activation of EGFR and Rac1	Reyes-Reyes et al., 2015
miR-199a-3p	K1 papillary thyroid carcinoma cells	Eventually involvement of HGF pathway(s)	Minna et al., 2014
Kalata B1 cyclotide	*Heterodera filipjevi*; nematode, juveniles, pre-parasitic second stage	Eventually involvement of ROS	Labudda et al., 2020
Methamphetamine	RA-differentiated SH-SY5Y neuroblastoma cells	Involving Ras–Rac1	Nara et al., 2010
MIPP	U251 GB cells	Targets endosomal trafficking involving Rab5 and Rab7	Overmeyer et al., 2011
MOMIPP	U251 and U373 GB cells; MCF-7 breast cancer cells; HMEC mammary epithelial cells; skin fibroblasts; HCT116 cells	Interference with glucose uptake; induction of JNK-mediated phosphorylation of Bcl-2 family members	Robinson et al., 2012; Trabbic et al., 2014, 2016; Cho et al., 2018; Colin et al., 2019; Li Z. et al., 2019; Oppong et al., 2020
Compound 13	MDA-MB-231, A375, HCT116, MCF-7 cancer cells		Huang et al., 2018
Isobavachalcone	NB4, U937, primary leukemic blast cells	Eventually AKT signaling	Yang et al., 2019
Azaindole-based compounds	MDA-MB-231 cells and other cells		Huang et al., 2018
Vacquinol-1	U-87 and #12537-GB glioma cells; rat GB models, RG2 and NS1; U373 GB cells	Involving MKK4 signaling; involving TRPM7 channels	Sander et al., 2017; Ahlstedt et al., 2018; Colin et al., 2019
Ursolic acid-derived compound 17	HeLa cervical cancer cells		Sun et al., 2017
Tubeimoside-1	SW480 colon carcinoma cells		Gong et al., 2018
Jaspine B	HGC-27 gastric cancer cells		Cingolani et al., 2017
Indolyl-pyridinyl-propenones; compounds 1a, 2b, 2p, 2q	U251 GB cells		Trabbic et al., 2015, 2016
Maduramicin	Primary chicken myocardial cells	Involving K-Ras–Rac 1 signaling	Gao et al., 2020
5-Iodoindole	*M. incognita*; *Bursaphelenchus xylophilus*; nematodes	Involving ROS	Rajasekharan et al., 2017, 2019, 2020
7-Iodoindole	*Bursaphelenchus xylophilus*		Rajasekharan et al., 2017
Abamectin	*Bursaphelenchus xylophilus*, nematode	Interaction with GluCL, disruption of osmoregulation	Rajasekharan et al., 2017
Extract of *Platycarya strobilacea* Sieb. et Zucc.	CNE1, CNE2 nasopharyngeal carcinoma cells	Involvement of Ras–MAPK signaling and c-Fos signaling; Rac1 activation	Zhu et al., 2017; Liu J. et al., 2020
Bacoside A from *Bacopa monnieri*	LN229 GB cells	CaMK2A phosphorylation, Ca^2+^ release from ER	John et al., 2017
Citreoviridin + Bortezomib	MCF7 human breast cancer cells	Inhibition of F1Fo ATP synthase (citreoviridin) + inhibition of 26S proteasome (bortezomib)	Chang et al., 2014
Abemaciclib	GBM neurospheres, glioma stem-like cells	Inhibition of CDK isoforms 4/6	Riess et al., 2021
Dinaciclib	GBM neurospheres, glioma stem-like cells	Inhibition of CDK isoforms 1/2/5/9	Riess et al., 2021
*s*-Triazine compound V6	U87 GB cells	Binding to vimentin	Zhang et al., 2021
CX-4945 (Silmitasertib)	SW-480, DLD-1, HT-29, HCT-116 colorectal cancer cells	Inhibition of CK2	Silva-Pavez et al., 2019
CX-4945 (Silmitasertib)	KKU-M213 cholangiocarcinoma cell line; eventually also in a wide range of cancer and immortalized cell lines	Independent of protein kinase CK2	Lertsuwan et al., 2018
CX-5011	HepG2 human hepatoma cells, zebrafish	Independent of protein kinase CK2	D’Amore et al., 2020
Graphene oxide (GO) nanoparticles	LO2 normal human hepatocytes	Eventually involving ROS-mediated cell stress following GO adherence and aggregation to the PM	Zheng et al., 2018
YM201636; Apilimod (STA-5326); Vacuolin-1; dominant-negative PIKfyve expression; wortmannin	COS7 African green monkey kidney cells, mouse embryonic fibroblasts; immortalized podocytes; B-cell non-Hodgkin lymphoma cells; C_2_C_12_ myoblasts; HEK 293 Human embryonic kidney cells, COS7 cells; NIH3T3 fibroblasts; CHO-T cells, 3T3L1 fibroblasts; U251 GB cells; HeLa cells	PYKFyfe inhibition; lack of active PIKFyfe; lack of Vps34 activity; inhibition of PI3Ks; vacuolation requires v-ATPase activity	Jefferies et al., 2008; Sbrissa et al., 2012, 2018; Compton et al., 2016; Sano et al., 2016; Saveanu and Lotersztajn, 2016; Gayle et al., 2017; Ikonomov et al., 2018; Li Z. et al., 2019
Rare earth oxide nanoparticles	HeLa cells	Leads to mTOR deactivation and nucleus translocation of TFEB	Lin et al., 2016
F14512	A549 non-small-cell lung cancer cells	Involves increased β-galactosidase activity	Brel et al., 2011
3Cpro	A549 and Calu-1 non-small-cell lung cancer cells	Dependent on v-ATPase, not dependent on Rac-1, Rab5, Rab7 or hyperstimulated macropinocytosis	Shubin et al., 2015
Antagomir knockdown of microRNA -103/107	HLEK cells, hTCEpi cells	Loss of microRNA-103/107; involvement of Src, Ras (no CD observed)	Park et al., 2016
Fenretinide	Rh4 and Rh30 alveolar rhabdomyosarcoma cells	EIPA-independent hyperstimulation of macropinocytosis; accumulation of early and late endosomal vacuoles; dependent on dynamin and ROS (new form of CD different form methuosis)	Brack et al., 2020
Epimedokoreanin C	NCI-H292 lung cancer cells	Involving Rac1 and Arf6	Liu X. et al., 2020
Hypertonicity	*Dictyostelium discoideum, axenically grown strain AX2*		Yuan and Chia, 2001
Knockdown of non-coding region of the C9orf72 gene		Altered of immune system genes expression; involving p53 activation, endothelin signaling; mitochondrial dysfunction; cellular glutamate accumulation; repression of mevalonate pathway; inhibition of isoprenylation; ROS formation	Fomin et al., 2018; Fomin, 2019

*Not yet termed methuosis; instead designated as “type 2 physiological cell death” (autophagic degeneration). For abbreviations and acronyms see list of abbreviations.

Figure 3 highlights major cellular aspects of the interrelation of (macro)pinocytosis, CVR, and VVR as discussed in this review.

## Perspectives

Understanding the mechanisms of pinocytosis and how it exerts its cell protective effects is critical to appreciate its diversity of physiological functions. It is also necessary to understand its dysfunctions and how they might lead to disease as well as to unravel how influencing pinocytosis may translate to potential applications for medical and other use.

For instance, designing of compounds like proteolytically stable peptidomimetics that are taken up by macropinocytosis, but which are able to escape from endosomes, could be used for intracellular biomolecular targeting (Yoo et al., 2020). Likewise, development of versatile drug carriers with a high loading capacity, such as nanoparticles, which are optimized for specific binding to cell surface receptors, and which are favoring both macropinocytic uptake and intracellular release by degradation of their shells, may open new ways for, e.g., cancer therapy. This principle was proven for hyaluronic acid-modified polymeric biodegradable mesoporous silica nanoparticles (Palanikumar et al., 2018).

Cancer cells frequently depend on autophagy to support their metabolic and energetic demands. However, inhibition of autophagy leads to compensatory stimulation of macropinocytosis to ensure cellular nutrient supply. This switch depends on the transcription factor NRF2, which is recruited to promoter regions of macropinocytosis-related genes. Recently, it has been shown that dual inhibition of autophagy and macropinocytosis is a successful strategy in treating mice with pancreatic ductal adenocarcinomas. Advancing this strategy to clinical applications may be promising for cancer therapy (Staff, 2021; Su et al., 2021).

Cell death by methuosis should meet special attention. The formation of huge cellular vacuoles, which eventually leads to cell death, is a frequently observed phenomenon in numerous pathologies and known for decades (Cameron, 1952; Henics and Wheatley, 1999). However, in many studies, the origin and mechanisms of the development of such cellular vacuoles were not or could not be determined. The term methuosis was coined in 2008 (Overmeyer et al., 2008). Hence, it will be necessary to carefully scrutinize the available body of literature for work describing methuosis-like phenomena and to eventually reevaluate it for better understanding the underlying mechanisms of deadly vacuolization and related types of cell death (Chi et al., 1999).

Recent studies indicate that volume regulation in cells and cell organelles is governed by the same biophysical principles and by the overlapping use of ion channels and transporters in distinct cellular compartments (Saric and Freeman, 2020, 2021; Chadwick et al., 2021a, b). That is, it starts with the asymmetric distribution of ions and materializes in the well-orchestrated uptake and processing of engulfed material in specialized vesicles (Chadwick et al., 2021a).

However, the contribution of inorganic ions may go beyond volume regulation. Inflammation and tissue injury are associated with a local extracellular increase in Ca^2+^ and enhancement of macropinocytosis in antigen-presenting cells (Canton, 2018). Presumably, the extracellular Ca^2+^ concentration is sensed by the calcium-sensing receptor (CaSR), which is expressed in myeloid cells (Canton, 2018). Accordingly, change in the extracellular ion composition may be critical in antigen processing and presentation *via* major histocompatibility complexes (MHCs) as well as recognition of non-self-antigens or modified endogenous substances by pattern recognition receptors (PRRs) (Canton, 2018). Consequently, failure in proper pinocytosis due to the formation of large vesicles could impair degradation of organic compounds or its delivery to PRRs or MHCs, which may have detrimental consequences in the immune response. The studies on CVR and VVR, pinocytotic activity, and delivery of compounds to MHCs or PRRs exemplify the power of ion gradients to understand physiology and pathophysiology of pinocytosis and methuosis, respectively. Despite Ca^2+^, other inorganic ions may have comparable dual effects on volume regulation and delivering of ligands to MHCs or PRRs. Thus, identification of ion channels and transporters required for establishing ion gradients could lead to a more detailed understanding of the complex interaction between distinct inorganic ions and cellular responses to tissue injuries or inflammation.

Beyond that, targeting specific ion transport mechanisms or their regulators will help to combat cellular entry of pathogens such as viruses or bacteria. For example, inhibition of PIKfyve, TPCs, NHEs, or the v-ATPase could help prevent cellular infection by Ebola virus, SARS-CoV-2, and other viruses (Mercer and Helenius, 2009; Sakurai et al., 2015; Kang et al., 2020; Mercer et al., 2020; Petersen et al., 2020; Spix et al., 2020; Eaton et al., 2021).

The number of compounds and materials that can induce or inhibit methuosis in various cell types and organisms is growing, and the fields of their use are just emerging. It will be necessary to unravel not only their specific modes of actions but also their harmful effects, as they might be used for a broad variety of applications, e.g., for eco-friendly biological pest control in agriculture to overcome multidrug resistance of parasites (Bogner et al., 2017; Rajasekharan and Lee, 2020; Rajasekharan et al., 2020). Importantly, they may emerge as new treatment options for various diseases, e.g., cancer (Song S. et al., 2020; Xiao et al., 2021), ALS, or dementia (Fomin et al., 2018; Fomin, 2019).

## Author Contributions

HK conceptualized the manuscript. HK, MR, and NB wrote and finalized the manuscript. NB captured and arranged the images displayed in Figure 1 and the accompanying Supplementary Video 1. MR prepared Tables 1, 2, and Figures 2, 3. All authors contributed to the article and approved the submitted version.

## Conflict of Interest

The authors declare that the research was conducted in the absence of any commercial or financial relationships that could be construed as a potential conflict of interest.

## Supplementary Material

Supplementary Video 1 Pinocytosis occurring in a multinucleated giant cell from a rat non-parenchymal hepatic cell line putatively representing immortalized monocytes (Kupffer cells). For explanations, see Figure 1 and text.

Supplementary Video 2 Close-up of Supplementary Video 1.

## Abbreviations

AA: arachidonic acid
Akt1: AKT serine/threonine kinase 1
ALS2: amyotrophic lateral sclerosis 2
ANO: anoctamin
APPL1: adaptor protein, phosphotyrosine interacting with PH domain and leucine zipper 1
AQP: aquaporin
Arf6: ADP-ribosylation factor 6
ARNO: ARF nucleotide binding-site opener
Arp2/3: actin-related protein 2/3
ASIC: acid-sensing ion channel
ALS2: alsin Rho guanine nucleotide exchange factor
ASOR: acid-activated outwardly rectifying chloride channel
ATP: adenosine triphosphate
AVD: apoptotic volume decrease
AVP: arginine vasopressin
BK channel: big-conductance Ca^2+^-activated K^+^ channel
CA: carbonic anhydrase
Ca^2+^_i_: intracellular Ca^2+^ concentration
CaMK: Ca^2+^/calmodulin-dependent kinase
Ca_v_: voltage-dependent calcium channel
Cav-1: caveolin-1
CD: cell death
CK: casein kinase
Cdc42: cell division control protein 42 homolog
CFTR: cystic fibrosis transmembrane conductance regulator
ClC: voltage-dependent chloride channel/exchanger
CLIC: chloride intracellular channel
3Cpro: 3C protease of human hepatitis A virus
CV: cell volume
CVR: cell volume regulation
EFA6: ARF6 guanine nucleotide exchange factor
ECF: extracellular fluid
EDRF: endothelium-derived relaxing factor
EGF: epidermal growth factor
EGFR: epdermal growth factor receptor
EIPA: 5-(*N*-ethyl-*N*-isopropyl)-amiloride
ENaC: epithelial Na^+^ channel
EET: 5 ′ -6 ′ -epoxyeicosatrienoic acid
ERK: extracellular signal-regulated kinase
Fig4: factor-induced gene 4
FRET: Förster/fluorescence resonance energy transfer
GAP: GTPase activating protein
GB: glioblastoma
GBM: glioblastoma multiforme
GEF: guanine nucleotide exchange factor
GluCL: glutamate-gated chloride channel
GLUT: glucose transporter
GPCR: see GPR
GPHR: Golgi pH regulator
GPR: G protein-coupled receptor
Grb2: growth factor receptor-bound protein 2
HLEK: human limbal keratinocytes
HGF: hepatocyte growth factor
hTCEpi: human limbal-derived epithelial cell line
HUVEC: human umbilical vein endothelial cell
ICF: intracellular fluid
ICl_swell_: swelling-activated Cl^–^ current
IGF-1: insulin like growth factor 1
IPMK: inositol polyphosphate multikinase
INO1: inositol-3-phosphate synthase
IMPase: inositol-1(or4)-monophosphatase
Ins(1,4,5)P3 or IP_3_: inositol 1,4,5-trisphosphate
IP: inositolphosphate
IP3K: inositol-trisphosphate 3-kinase
IP3R: IP_3_ receptor
IPP: inositol-1,4 biphosphate 1-phosphatase
INFγ: interferon gamma
INPP5: inositol-polyphosphate 5-phosphatase
INPase: inositol-phosphate phosphatase
INPP: inositol-1,4-bisphosphate 1-phosphatase
INPP5: inositol polyphosphate 5 phosphatase
IPPK: inositol-pentakisphosphate 2-kinase
ITPK: inositol 1,3,4-trisphosphate 5/6 kinase, acts also as inositol polyphosphate phosphatase
K_v_: voltage-gated potassium channel
LPS: lipopolysaccharide
LTD4: leukotriene D4
mAB: monoclonal antibody
MAPK: mitogen-activated protein kinase
M-CSF: macrophage colony-stimulating factor
MHC: major histocompatibility complex
MINPP: multiple inositol-polyphosphate phosphatase
MIPP: 3-(2-methyl-1H indol-3-yl)-1-(4-pyridinyl)-2-propen-1-one
miR: microRNA
MKK4: mitogen-activated protein kinase kinase 4
MLC1: megalencephalic leukoencephalopathy with subcortical cysts 1
MOMIPP, 3-(5-methoxy: 2-methyl-1H-indol-3-yl)-1-(4-pyridinyl)-2-propen-1-one
MTM1: myotubularin 1
MTMR: myotubularin-related protein
MTMR6: myotubularin R6
mTOR: mammalian target of rapamycin
MVB: multi-vesicular body
NAADP: nicotinic acid adenine dinucleotide phosphate
NCX: Na^+^/Ca^2+^-exchanger
NFAT5: nuclear factor of activated T cells 5
NGF: nerve growth factor
NOSTRIN: nitric oxide synthase trafficking inducer
NRF2: nuclear factor erythroid 2-related factor 2
NVI: necrotic volume increase
OCR: Lowe oculocerebrorenal syndrome protein
Orai: named after the keepers of the gates of heaven in Greek mythology
synonym: CRACM: calcium release-activated calcium channel protein
OMIM: Online Mendelian Inheritance in Man^®^
Ostm1: osteoclastogenesis-associated transmembrane protein
p53: tumor protein P53 or cellular tumor antigen p53
Pak: p21-activated kinase
Panx1: pannexin 1
PDGF: platelet-derived growth factor
PDK1: 3-phosphoinositide-dependent protein kinase 1
PEG: polyethyleneglycol
Pf: osmotic water permeability
PFKFB3: 6-phosphofructo-2-kinase/fructose-2,6 biphosphatase 3
PH: pleckstrin homology
pH_i_: intracellular pH
PI: phosphoinositide or phosphatidylinositol
PI(3)P: phosphatidylinositol 3-phosphate
PI(3,4,5)P3: phosphatidylinositol (3,4,5)-trisphosphate
PI(4,5)P2: phosphatidylinositol 4,5-bisphosphate
PI3K: phosphatidylinositol 3 kinase
PIKfyve: phosphoinositide kinase FYVE-type zinc finger containing
PKB: protein kinase B
PLA2: phospholipase A2
PLC: phospholipase C
PM: plasma membrane
PS: phosphatidylserine
PRR: pattern recognition receptor
PTEN: phosphatase and tensin homolog
P2X-R: ATP-gated P2X receptor cation channel
RA: retinoic acid
Rab: Ras-related in brain
Rac: Ras-related C3 botulinum toxin substrate
RACK1: receptor for activated C kinase 1
Raf1: rapidly accelerated fibrosarcoma 1
Rap1: Ras-proximate-1/Ras-related protein 1
Ras: rat sarcoma
Rasal: Ras-GTPase–activating-like protein
RGBARG: RCC1-RhoGEF-BAR-and-RasGAP-containing protein
Rheb: Ras homolog enriched in brain
Rho: Ras homologous
RIN1: Ras and Rab interactor 1
RN-tre: Related to the N-terminus of tre
ROS: reactive oxygen species
RVD: regulatory volume decrease
RVI: regulatory volume increase
SGK: serum- and glucocorticoid-inducible protein kinase
SLC: solute carrier
SMIT: Na^+^/myo-inositol transporter
SOCE: store-operated calcium entry
STIM1: stromal interaction molecule 1
TFEB: transcription factor EB
TMEM: transmembrane protein
TonEBP: tonicity responsive enhancer (TonE) binding protein
TORC: target of rapamycin complex
TPC: two-pore channel
TRP: transient receptor potential
TRPML: TRP channel of mucolipin subfamily
TRPM7: Transient receptor potential cation channel subfamily M member 7
TSC: tuberous sclerosis complex
Ttyh: Tweety homolog
v-ATPase: vacuolar H^+^-ATPase
Vps34: vacuolar protein sorting 34
VSOR: volume-sensitive outwardly rectifying Cl^–^ channel/current
VRAC: volume-regulated anion channel/current
VVR: vesicular volume regulation
WASP: Wiskott–Aldrich syndrome protein
WNK: with no lysine kinase
